# Autosomal DIPs for population genetic structure and differentiation analyses of Chinese Xinjiang Kyrgyz ethnic group

**DOI:** 10.1038/s41598-018-29010-8

**Published:** 2018-07-23

**Authors:** Yuxin Guo, Chong Chen, Xiaoye Jin, Wei Cui, Yuanyuan Wei, Hongdan Wang, Tingting Kong, Yuling Mu, Bofeng Zhu

**Affiliations:** 1 Key Laboratory of Shaanxi Province for Craniofacial Precision Medicine Research, College of Stomatology, Xi’an Jiaotong University, Xi’an, 710004 P. R. China; 2 Clinical Research Center of Shaanxi Province for Dental and Maxillofacial Diseases, College of Stomatology, Xi’an Jiaotong University, Xi’an, 710004 P. R. China; 3 College of Medicine & Forensics, Xi’an Jiaotong University Health Science Center, Xi’an, 710061 P. R. China; 4 Medical Genetics Institute of Henan Province, Henan Provincial People’s Hospital, Zhengzhou University People’s Hospital, Zhengzhou, 450003 P. R. China

## Abstract

In recent years, deletion and insertion polymorphisms (DIPs) were treated as a novel complementary tool with huge potential for forensic applications. In this study, we utilized 30 DIP loci to make a comprehensive research of allele frequency distribution and compute forensic parameters to evaluate the efficiency of forensic applications in the 295 unrelated healthy individuals of Kyrgyz group, and in addition, infer the genetic relationships between Kyrgyz group and 24 other previously studied groups. No significant departures from Hardy-Weinberg equilibrium and linkage disequilibrium were observed at these 30 DIP loci. The combined power of discrimination and the combined probability of exclusion for all 30 DIP loci in Kyrgyz group were 0.9999999999989 and 0.9939, respectively. Furthermore, the results of the interpopulation differentiations, phylogenetic reconstruction, population genetic structure and principal component analyses suggested that Kyrgyz group had relatively close genetic relationships with Kazakh and Uygur groups. However, it was also important to stress that 15 loci were selected out from these 30 DIP loci using the method of selecting ancestry markers, which could be utilized for further ancestry inference study relatively.

## Introduction

The majority of human genome sequence variation could be attributable to nucleotide substitution polymorphisms, with the rest attributable to deletion and insertion polymorphisms (DIPs)^1^. DIPs could in turn be split into those with multiple alleles (multiallelic) and with only two alleles (diallelic)^1^. Nearly all of the multiallelic DIPs were based on tandem repeats, mostly short tandem repeats (STRs)^1^, however, the 30 DIP loci chosen in this study were diallelic.

Further, it was particularly noteworthy that DIPs possessed the desirable properties of both STRs and single nucleotide polymorphisms (SNPs), which could be summarized as followings: (i) length polymorphisms allowing them amenable to analyze through simple capillary electrophoresis in common forensic DNA laboratories^2^; (ii) an abundance of distributions in human genome with the density ranking only second to that of SNPs^3^; (iii) small amplicon size improving the probabilities of successfully analyses for highly degraded DNA^4^; (iv) lower rates of mutations, which made them more stable than STRs^2^; (v) PCR amplification without the generation of stutter peaks, making the allelic genotyping results more concise and precise^5^; (vi) the marked differences of allele frequencies in some loci between diverse populations from geographically separated regions, therefore they had potential to be applied in biogeographic ancestry analyses^6,7^.

The Kyrgyz ethnic minority with the population totaling over 0.18 million belongs to the 56 ethnic groups officially published by the People’s Republic of China^8^, which are mainly found in the southwestern part of the Xinjiang Uygur Autonomous Region, China^8^. Now we use Qiagen Investigator DIPplex reagent (Qiagen, Hilden, Germany), a commercial kit, to analyze 30 DIP loci distributed on 19 pairs of chromosomes and in addition, this kit was put into use in the previous population studies which were already published^9–11^. We gathered the bloodstain samples of Kyrgyz group in Xinjiang Uygur Autonomous Region and used the kit mentioned above to obtain population data to acquire more information about the Kyrgyz ethnic minority’s genetic background.

## Results and Discussion

### The analyses of allelic frequency distributions and forensic parameters

**Figure 1:**
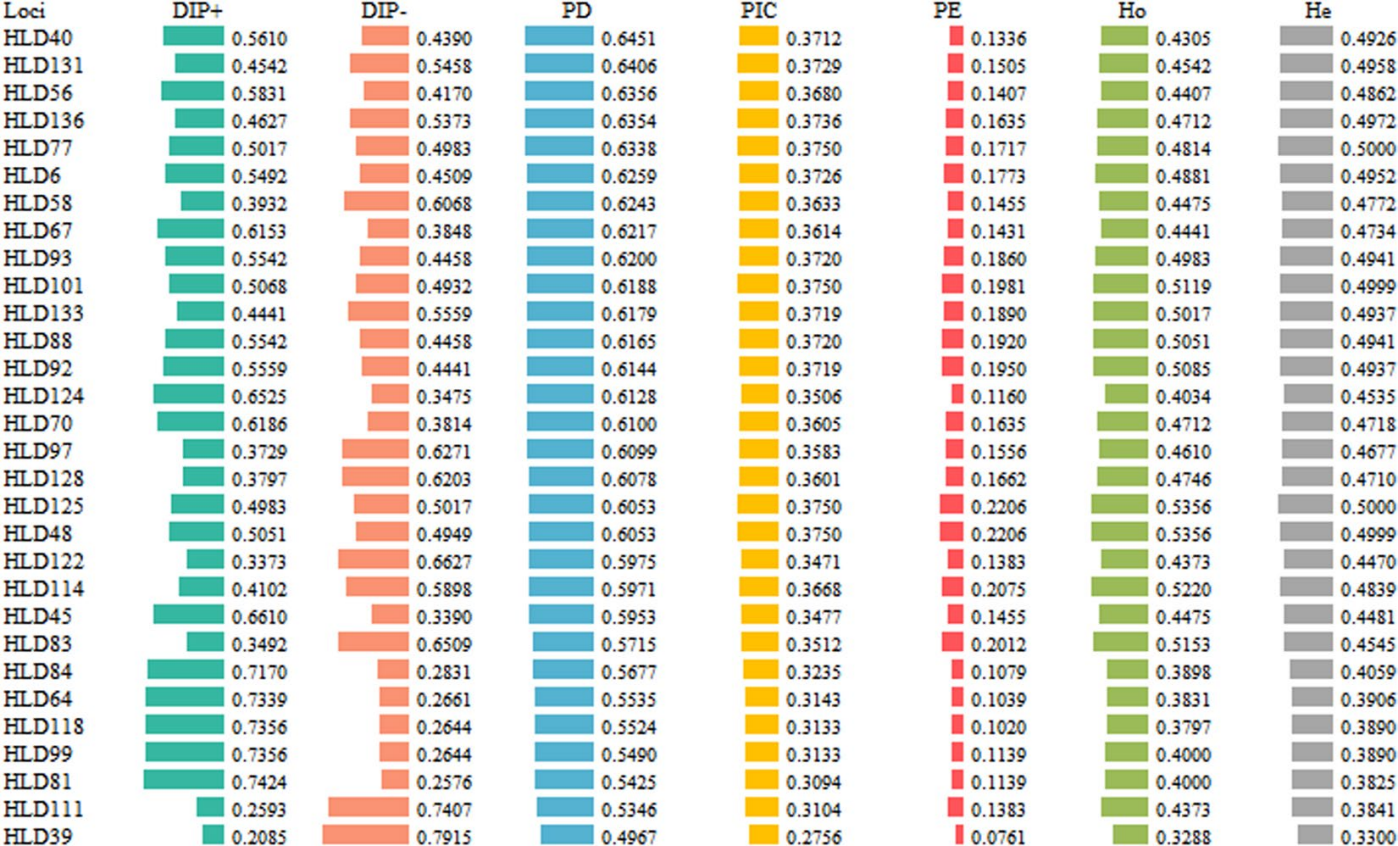
Plots of allele frequencies and forensic parameters were mapped on account of 30 DIP loci in the Chinese Xinjiang Kyrgyz group.

The systematically experimental operations and analyses of the samples had been conducted under the laboratory stringent criteria before the data of Kyrgyz group obtained. There were no significant departures from Hardy-Weinberg equilibrium (HWE) in the 30 DIPs after applying a Bonferroni correction (*p* = 0.05/30 = 0.0017). Allele frequencies and forensic efficiency parameters of 30 DIPs in Kyrgyz group were depicted in Fig. 1. The expected heterozygosity (He) values ranged from 0.3300 (HLD39) to 0.5000 (HLD77 and HLD125) and the observed heterozygosity (Ho) values varied from 0.3288 (HLD39) to 0.5356 (HLD48 and HLD125). The values of polymorphic information content (PIC) were in the range of 0.2756 to 0.3750 with a mean value of 0.3524. Additionally, the maximum value of power of exclusion (PE) was 0.2206 at HLD48 and HLD125 loci, whereas the minimum was 0.0761 at HLD39 locus. The combined probability of exclusion (CPE) for all 30 DIP loci in Kyrgyz group was 0.9939. However, the CPE value was relatively low (compared with which of STRs^12^) implying that the panel of 30 DIP loci could be a complementary tool for STR typing system in forensic paternity cases. We also detected the power of discrimination (PD) ranging from 0.4967 (HLD39) to 0.6451 (HLD40), and combined power of discrimination (CPD) reached 0.9999999999989, which was able to meet the satisfactory levels for the individual identification of forensic demands^13^. Among these forensic parameters, it was significantly pronounced that the lowest values of He, Ho, PD, PE, PIC were obtained at HLD39 locus, indicating that HLD39 locus showed relative low forensic efficiency in the studied Kyrgyz ethnic group.

In addition, the values of minor allele frequency (MAF) of 0.0700 to 0.2000 were found at 12 loci (Supplementary Table 1), including HLD39, HLD48, HLD58, HLD81, HLD83, HLD84, HLD99, HLD111, HLD114, HLD122, HLD125 and HLD128 loci. In this study, the MAF values of some loci were generally low, indicating that the panel of 30 DIP loci might have great potential to detect population structure and analyze population genetic relationships^14^. Therefore, to confirm our hypothesis, we did the following analyses of 25 populations based on these 30 DIP loci to explore the population origin and genetic structure of Xinjiang Kyrgyz.

### Linkage disequilibrium analyses

Linkage disequilibrium (LD) tests among these 30 DIP loci in Kyrgyz group were performed using the SNPAnalyzer program. As shown in the Supplementary Figure 1, the pairwise LD analyses indicated that no significant LD with the coverage of thick black curve existed in the plot, showing that these 30 DIP loci were independent with each other in the studied Kyrgyz ethnic group.

### Interpopulation differentiations

**Figure 2:**
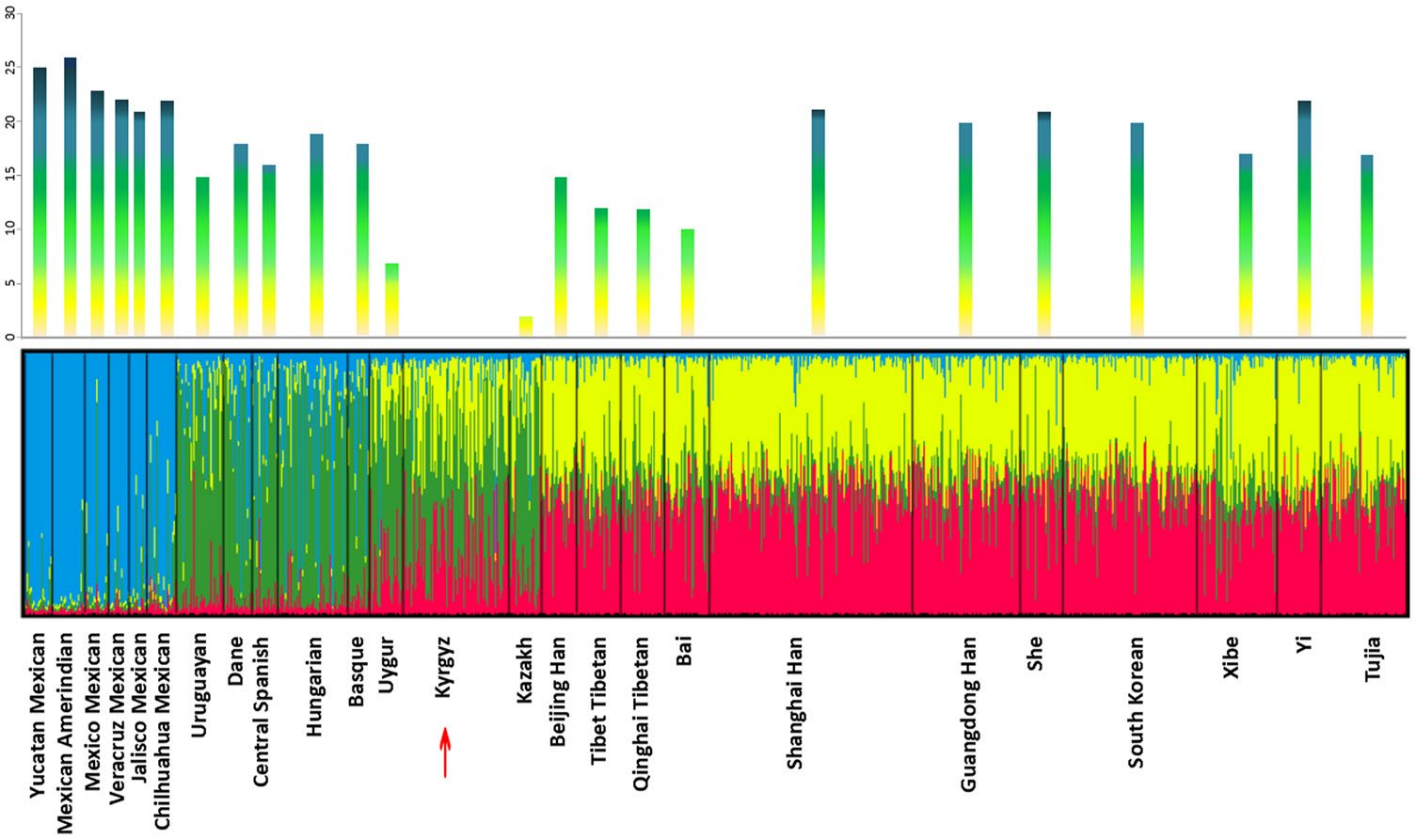
Clustering structure for the full-loci dataset assuming *K* = 4 of the 25 groups combined with the bar diagram representing various numbers of pairwise *p*-value with significant differences (*p* < 0.05).

To explore the hereditary similarities and differences, the studied Kyrgyz group was compared with previously published groups at these 30 DIP loci utilizing the analysis of molecular variance (AMOVA) method on the basis of Arlequin software version 3.1. The locus-by-locus *p* values were shown in Supplementary Table 2 and the number of loci with significant differences (*p* < 0.05) between Kyrgyz and the 24 reference populations were presented intuitionally in bar diagram format combined with the result of structure analysis (*K* = 4) in Fig. 2. Statistically significant differences were detected between the studied Kyrgyz group and Kazakh group^11^ at two loci; Uygur group^11^ at seven loci; Bai group^15^ at ten loci; Tibet Tibetan and Qinghai Tibetan group^16^ at 12 loci; Beijing Han^11^ and Uruguayan group^17^ at 15 loci; Central Spanish group^18^ at 16 loci; Tujia^19^ and Xibe group^20^ at 17 loci; Basque^18^ and Dane group^21^ at 18 loci; Hungarian group^22^ at 19 loci; Guangdong Han^23^ and South Korean group^24^ at 20 loci; Shanghai Han^25^ and She group^25^ at 21 loci; Yi group^26^ at 22 loci; and six Mexican groups^27^ at 21–26 loci, respectively. According to the diagram, the Kyrgyz group had the lowest genetic divergence with Kazakh group (significant differences found at two loci) in contrast with Mexican Amerindian group (significant differences found at 26 loci). Furthermore, some loci with high ethnic diversities could be observed: two loci (HLD81 and HLD111) all showed significant differences at 21 compared groups, on the contrary, HLD101 and HLD88 at four and six respectively. It was suggested that the abilities of some DIP loci to distinguish ethnic groups were at different levels^3^. Note that, studies of more DIP loci in more ethnic populations should be required for the different application purposes in forensic science.

**Figure 3:**
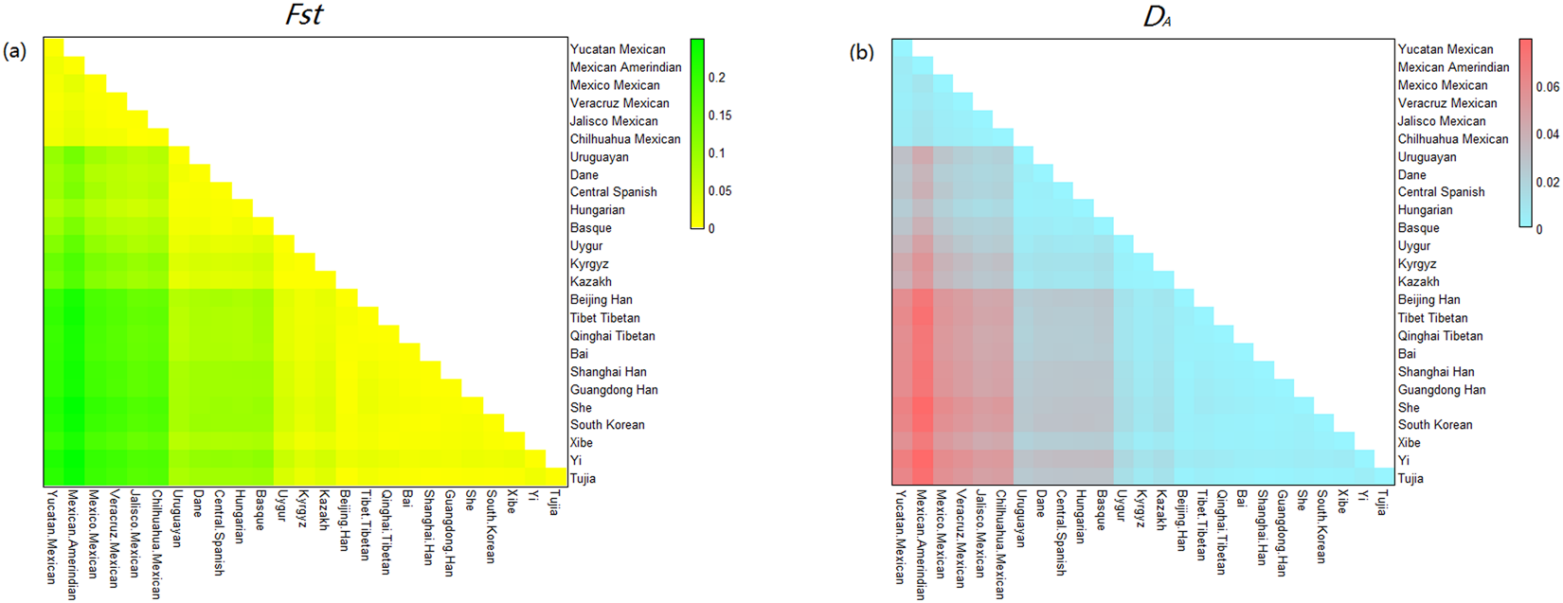
(**a**) Heat map of pairwise *Fst* values of 30 DIP loci in Xinjiang Kyrgyz and 24 previously studied populations based on *R* software. (**b**) Heat map of pairwise *D*_*A*_ values of 30 DIP loci among Xinjiang Kyrgyz and 24 previously published populations conducted with *R* software.

In addition, we conducted two heat maps using *R* statistical software. Based on 30 DIP loci, one heat map of pairwise fixation index (*Fst*) values (Supplementary Table 3) calculated by GENEPOP program was labeled on Fig. 3a, revealing the genetic differentiations among the studied Kyrgyz group and 24 reference populations. *Fst* is directly related to the variance in allele frequency among populations. The larger *Fst* value is, the higher genetic divergence between pairwise populations is, and vice versa^28^. As presented in Fig. 3a, the deeper green color stood for the larger *Fst* value, which meant the more differentiation existed between pairwise populations; conversely, the deeper yellow color meant the smaller *Fst* value as well as the less differentiation^28^. We could also detect intuitively that the 25 studied populations could be separated into four clusters based on the depth of color: the Mexican groups, European groups and Uruguayan, Central Asian groups, and East Asian groups. Focusing on the Central Asian groups, we could come to a conclusion that the studied Kyrgyz had lower *Fst* values with Kazakh and Uygur groups. For further detail, the studied Kyrgyz had deeper yellow color with Kazakh group rather than Uygur group, which indicated Kyrgyz group might have a closer genetic relationship or more similar origin with Kazakh group.

For further elucidation of the Chinese population affiliations, another heat map exhibited in Fig. 3b plotted based on pairwise *D*_*A*_ values (Supplementary Table 4) which were carried out with DISPAN program, showing the various genetic distances among the studied Kyrgyz and 24 reference populations. The deeper red color represented the greater *D*_*A*_ value meant the bigger genetic distance, while the deeper blue color meant the smaller *D*_*A*_ value along with the closer genetic distance. In addition, each pairwise population with a closer genetic distance also had a smaller genetic divergence. In this study, small *D*_*A*_ value with deep blue color were also found in four clusters: the Mexican groups, European groups and Uruguayan, Central Asian groups, and East Asian groups, which was consistent with the result of *Fst* heat map. On the purpose of more direct analyses, a bar chart of both *Fst* and *D*_*A*_ values between the studied Kyrgyz group and 24 reference populations were shown in the Supplementary Figure 2, respectively, displaying the high consistency of trend between both kinds of values. It was evident that the studied Kyrgyz group had the shortest genetic distance with the Central Asian groups (Kazakh and Uygur groups), which meant that these three groups with small genetic divergence mentioned above might have similar consanguineous relationships to some extent.

### Principal component analysis

**Figure 4:**
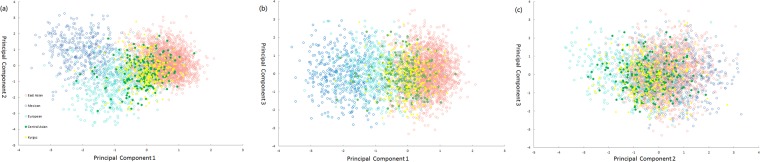
The PCA plots were analyzed at individual level using 30 DIP loci. (**a**) based on PC1and PC2 (**b**) based on PC1 and PC3 (**c**) based on PC2 and PC3. The red dots represented for East Asians, deep blue dots for Mexicans, light blue dots for Europeans, green dots for Central Asians, and yellow dots for Kyrgyzs.

The genetic relationships between Kyrgyz group and other 24 populations were presented by three plots of principal component analysis (PCA) utilizing the SPSS 18.0 software (SPSS, Chicago, IL, USA). As shown in Fig. 4a, 25 populations were divided into four colored clusters based on PC1 (9.844%) and PC2 (4.373%), including Central Asian groups (green) without Kyrgyz group, East Asian groups (pink), six Mexican (deep blue) and European groups (light blue). Then, Kyrgyz group was represented by yellow points, with one yellow dot standing for an individual. Nevertheless, Fig. 4c based on PC2 (4.373%) and PC3 (3.698%) was in a blended cluster with small capacity to discriminate each continent apart contrasting with Fig. 4a and in addition, Fig. 4b on the basis of PC1 (9.844%) and PC3 (3.698%) had a relatively limited discrimination between Fig. 4a,c.

As presented in Fig. 4a, all individuals from 25 populations were partitioned into four main regions keeping in line with their intercontinental distributions roughly, and individuals from Kyrgyz, Kazakh and Uygur groups were scattered between East Asians and Europeans as expected, conforming to the previous studies and ethnic migration records^29,30^. Since Western Han Dynasty to the middle of the Qing Dynasty, mainly from the Yenisai River to the Tianshan Mountains and Central Asia, Kyrgyz group experienced five westward migrations which were basically facilitated by warfare^29^. The studied Kyrgyz group which inhabits the southwestern region of Xinjiang broadly assimilated Western Regions culture after the long term of mixed dwelling with the Uygurs, Kazakhs, Hans and Mongolians etc^29^. In contrast, the ancestry of Xinjiang Xibe is different from Kyrgyz group. Xibe traditionally resided in northeast China and immigrated to Xinjiang during the middle of the eighteenth century. Thus, Xinjiang Xibe group had the same pattern with other East Asian groups as shown in structure analysis of Fig. 2, displaying the same cluster pattern as previously reported^31^. That was the reason why the Xinjiang Xibe group came from the same region as Kyrgyz group was treated as a member of East Asian groups in Fig 4. The above result also showed the close genetic relationships between the Kyrgyz, Kazakh and Uygur groups, and implied that Kyrgyz group, in this study, might play an important role in culture exchange and gene flow between East Asians and Europeans^8^.

### Multidimentional scaling analysis

**Figure 5:**
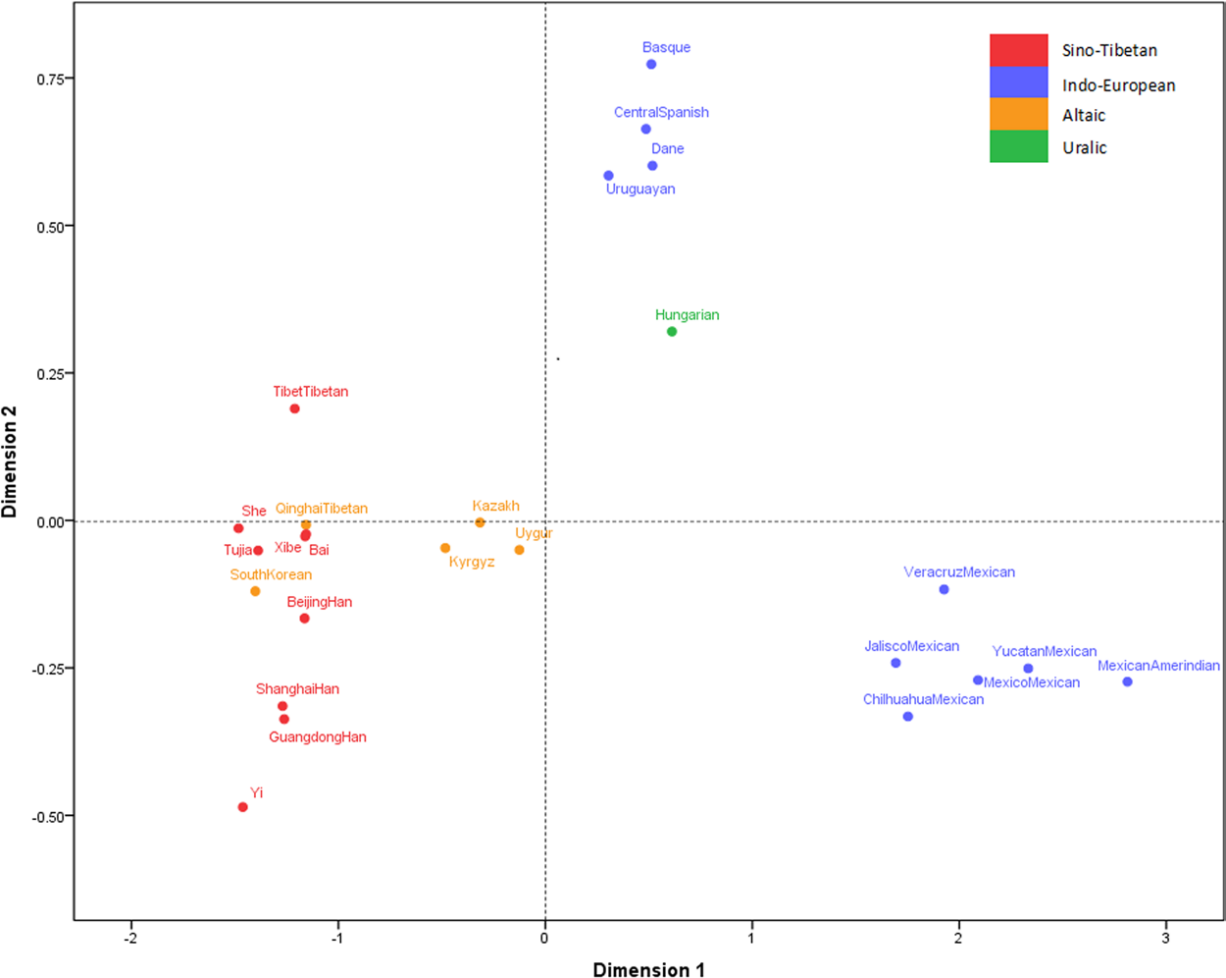
MDS analysis of Xinjiang Kyrgyz group and other 24 reference populations were conducted by SPSS18.0 based on pairwise *Fst* values.

For further investigation of genetic correlations among 25 populations, multidimentional scaling (MDS) analysis was performed using SPSS 18.0 software (SPSS, Chicago, IL, USA) and provided a two-dimensional representation of genetic relationships based on pairwise *Fst* values calculated by GENEPOP program. As shown in Fig. 5, each dot in the two dimensional space indicated one population and it was given a color according to language family that the population belonged to. The various distances between different dots showed different genetic relationships among the populations. In detail, the closer the two dots were, the closer the genetic relationships they had. In the light of various distances between dots, 25 populations mentioned above were divided into four clusters roughly: the East Asian groups, the Mexican groups, European groups and Uruguayan, and Central Asian groups, which was in concordance with their geographic distributions.

### Phylogenetic analysis

**Figure 6:**
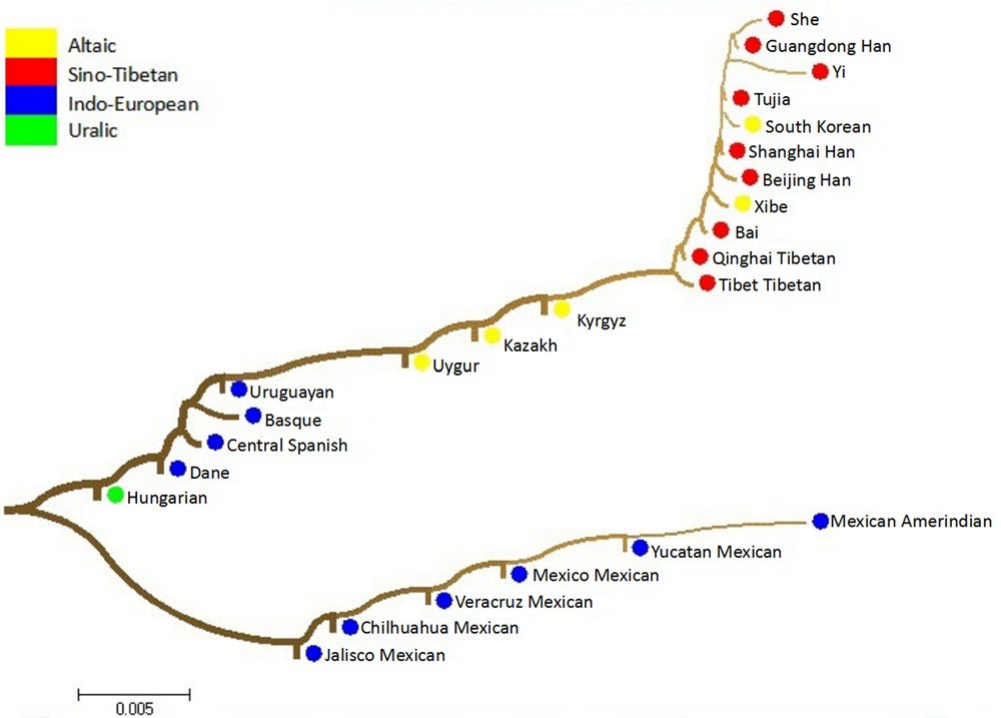
Phylogenetic tree constructed by the neighbour joining method based on the *D*_*A*_ distances among the 25 populations.

In order to estimate the studied population affiliations, based on the *D*_*A*_ values, phylogenetic reconstruction tree encompassing two main branches was performed to verify the genetic relationships between the Xinjiang Kyrgyz and 24 reference populations, which was reconstructed utilizing neighbor joining method by MAGA software v5.0. As shown in Fig. 6, two main branches could be clearly identified in the phylogenetic tree. The upper branch was composed of three clusters: eleven East Asian groups on the upper-right side including South Korean and most Chinese populations such as Tibetan populations from two different regions, Han populations from three different regions, She, Yi, Tujia, Xibe and Bai groups; Uruguayan group and four European groups on the upper-left side; and Central Asian groups (Kazakh, Kyrgyz and Uygur groups) in the middle of the upper branch. The lower branch merely consisted of six Mexican populations. The clustering results of dendrogram focusing on the Xinjiang Kyrgyz, Kazakh and Uygur populations roughly congruent with the former analyses such as the results of PCA and MDS analysis.

In long-term development of Chinese history, the Kazakh group was formed as a combination of the Turkic, Wusun, Khitan and Mongolian people, and Uygur group was stemmed from a branch of Turkic people^11^, while Kyrgyz group partially mixed the genetic component of Mongol, Khitan, Turkic, Uygur and Han groups since the ancient time^29,32^. Furthermore, Kyrgyz, Kazakh and Uygur groups almost possessed the same religious belief and the close geographic distance, more likely leading to the cultural exchange among these three groups^29^.

### Clustering analyses

To assess the population stratification and calculate the proportion of different ancestry components in various populations, the structure analyses based on the genotyping data of the studied Kyrgyz group and 24 reference populations were conducted with Structure 2.3.4 software which could infer individual genetic ancestry coefficients by controlling the values of *K* that represented the number of hypothetical ancestral populations^33^. As shown in Supplementary Figure 3, 25 populations were separated by black lines and each single vertical line represented one individual was partitioned into several colored segments on behalf of the individual’s estimated membership fractions^34^. At *K* = 2, the East Asian groups were distinguished from both European groups (including Uruguayan, Dane, Central Spanish, Basque and Hungarian groups) and Mexican groups (including Yucatan Mexican, Mexican Amerindian, Mexico Mexican, Veracruz Mexican, Jalisco Mexican and Chilhuahua Mexican groups), with the constitution of entirely red components. At the same time, the Central Asian groups, including Kyrgyz, Kazakh and Uygur groups, shared obviously mixed memberships in red and green color. Five European groups and six Mexican groups were almost filled with green components; therefore, European and Mexican groups could not separate from each other at *K* = 2. Whereas, the Central Asian groups were separated from other populations evidently with the combination of red, green and yellow components in different proportions at *K* = 4 (Fig. 2), which was verified as the most suitable *K* value relying on the output posterior probability results^35^. As a result, genetic clusters were roughly in accordance with collections of geographically similar populations^34^.

Based on the analyses mentioned above, we have a sufficient reason to insist that some loci with great genetic divergence were existed in these 30 DIP loci, which were valid for detecting population structure and distinguishing Kyrgyz ancestry information from other populations distributed in different administrative divisions. However, abilities of these 30 DIP loci appeared to be diverse at the power of describing population clusters. In order to pinpoint the loci that more contributed to population discrimination, 15 loci among these 30 DIP loci, including HLD39, HLD45, HLD48, HLD56, HLD58, HLD70, HLD64, HLD81, HLD83, HLD111, HLD114, HLD118, HLD122, HLD125 and HLD128, were selected out based on population-specific allele frequencies (δ values >0.29)^36^, using the method of selecting ancestry information markers (AIMs) that discrepant values of average insertion allele frequencies among the clusters (four clusters as detected above: the Mexican groups, European groups and Uruguayan, Central Asian groups, and East Asian groups) could be over 0.29^37^. After that, we again performed structure analyses of the studied Kyrgyz group and 24 reference populations with the result (*K* = 4) showing in the Supplementary Figure 4b, significantly contrasting with Fig. 4a which was performed by the rest eliminated 15 loci. We could draw the same conclusion in the structure Fig. 4b that 25 populations were partitioned into four clusters roughly, three Central Asian groups and compared with 11 East Asian groups, five European populations and six Mexican populations, the studied Kyrgyz group had more intimate membership with Kazakh and Uygur groups^38^. However, in the Fig. 4a, the population stratification could hardly be detected, which indicated the ability of ancestry inference of the rest eliminated 15 loci was relatively insufficient.

In brief, to explore the genetic background and genetic structure of the studied Kyrgyz and other populations further, we could choose efficient AIMs with reference to the method mentioned above to acquire more comprehensive and accurate population genetic information and to lay a solid foundation for the ancestry inference study in the future.

## Conclusion

In this study, the allele frequencies and statistically forensic parameters of the autosomal 30 DIP loci were obtained for the researches of population genetics and forensic applications. The panel, as a useful forensic tool, was suit for individual identification, but could barely be treated as supplementary markers for STR loci in paternity testing. The results of interpopulation differentiations, genetic distances, principal component, multidimentional scaling, phylogenetic and structure analyses indicated close genetic relationships between Kyrgyz and the two Central Asian groups (Kazakh and Uygur groups). Furthermore, we selected out 15 loci with sufficient capacity of ancestry inference from these 30 DIP loci, which could be implemented in ancestry inference study. For the sake of better understand the origin and genetic evolution of Kyrgyz group, further study should be performed in later research.

## Material and Methods

### Sample collections and DNA extraction

The bloodstain samples of 295 unrelated healthy individuals were collected from Kyrgyz group residing in Xinjiang Uygur Autonomous Region, China. During the course of collecting samples, we excluded the samples gathered from two individuals who had blood relationships within three generations. All participants concerned to this study provided the written informed consents. The research was in accordance with the human and ethical research principles and approved by the ethics committee of Xi’an Jiaotong University Health Science Center.

### PCR amplification and DIP genotyping

On GeneAmp PCR System 9700 thermal cycler (Applied Biosystems, Foster City, CA, USA), a multiple PCR amplification with five-color fluorescence of autosomal 30 DIPs was performed with Investigator DIPplex reagent on the basis of manufacturer’s instructions. Genotyping of DIPs was analyzed by capillary electrophoresis on ABI 3500 Genetic Analyzer (Applied Biosystems, Foster City, CA, USA) and processed by GeneMapperv3.2 software (Applied Biosystems, Foster City, CA, USA).

### Quality control

The study was conducted following ISFG recommendations on the analysis of the DNA polymorphisms as described by Schneider^39^.

### Statistical analyses

Forensic statistical parameters of 30 DIP loci in Kyrgyz group, such as the values of HWE, Ho, PE, PD, PIC and allele frequency distributions were calculated by modified Powerstate (version1.2) spreadsheet (Promega, Madison, WI, USA), and He values were calculated based on allele frequencies. Arlequin software (version3.0)^40^ was chosen to evaluate locus-by-locus *p* values using AMOVA method. The heat maps were performed by *R* statistical software v3.0.2^41^ based on *Fst* and *D*_*A*_ values which were calculated by allele frequencies and raw population data, respectively. The SNPAnalyzer (version2.0 Istech, South Korea)^42^ was selected to test LD for all pairwise DIP loci. Three plots of PCA and a plot of MDS analysis were carried out by SPSS 18.0 software (SPSS, Chicago, IL, USA). Population genetic structure analyses were conducted by STRUCTURE v2.2 program^33^. The phylogenetic tree was described using MAGA v6.06 software based on *D*_*A*_ values calculated by DISPAN program^43^.

### Data availability

The datasets analyzed during the current study are available from the corresponding author upon reasonable request.

## Electronic supplementary material

Supplementary Information
