# Supplementary material for: Autosomal DIPs for population genetic structure and differentiation analyses of Chinese Xinjiang Kyrgyz ethnic group
Source: Sci Rep. 2018 Jul 23;8:11054. doi: 10.1038/s41598-018-29010-8 (PMC6056483; doi:10.1038/s41598-018-29010-8)
Supplement: Supplementary file 1 — Supplementary Information [file 41598_2018_29010_MOESM1_ESM.docx]

**Title**

Autosomal DIPs for population genetic structure and differentiation analyses of Chinese Xinjiang Kyrgyz ethnic group

Yuxin Guo^1,2,3^, Chong Chen^1,2,3^, Xiaoye Jin^1,2,3^, Wei Cui^1,2,3^, Yuanyuan Wei^1,2^, Hongdan Wang^4^, Tingting Kong^1,2^, Yuling Mu^3^, Bofeng Zhu^1,2,3,5^

*^1^Key Laboratory of Shaanxi Province for Craniofacial Precision Medicine Research, College of Stomatology, Xi’an Jiaotong University, Xi’an 710004, China*

*^2^Clinical Research Center of Shaanxi Province for Dental and Maxillofacial Diseases, College of Stomatology, Xi’an Jiaotong University, Xi’an 710004, China*

*^3^College of Medicine & Forensics, Xi’an Jiaotong University Health Science Center, Xi’an 710061, P. R. China*

*^4^Medical Genetics Institute of Henan Province, Henan Provincial People’s Hospital, Zhengzhou University People's Hospital, Zhengzhou 450003, P. R. China*

*^5^Department of Forensic Genetics, School of Forensic Medicine, Southern Medical University, Guangzhou 510515, P. R. China*

*Correspondence to: Bofeng Zhu, email: zhubofeng7372@126.com*

| Supplementary Table 1. Allele frequencies of the studied Kyrgyz and 24 reference populations. | | | | | | | | | | | | | | | | | | | | | | | | | | | | | | | | | | | | | | | | | | | | |  |  |
| --- | --- | --- | --- | --- | --- | --- | --- | --- | --- | --- | --- | --- | --- | --- | --- | --- | --- | --- | --- | --- | --- | --- | --- | --- | --- | --- | --- | --- | --- | --- | --- | --- | --- | --- | --- | --- | --- | --- | --- | --- | --- | --- | --- | --- | --- | --- |
| Populations | | HLD6 | |  | HLD39 | |  | HLD40 | |  | HLD45 | |  | HLD48 | |  | HLD56 | |  | HLD58 | |  | HLD64 | |  | HLD67 | |  | HLD70 | |  | HLD77 | |  | HLD81 | |  | HLD83 | |  | HLD84 | |  | HLD88 | | |
|  |  | DIP+ | DIP- |  | DIP+ | DIP- |  | DIP+ | DIP- |  | DIP+ | DIP- |  | DIP+ | DIP- |  | DIP+ | DIP- |  | DIP+ | DIP- |  | DIP+ | DIP- |  | DIP+ | DIP- |  | DIP+ | DIP- |  | DIP+ | DIP- |  | DIP+ | DIP- |  | DIP+ | DIP- |  | DIP+ | DIP- |  | DIP+ | DIP- | |
| Yucatan Mexican | | 0.4342 | 0.5658 |  | 0.5724 | 0.4276 |  | 0.4408 | 0.5592 |  | 0.3421 | 0.6579 |  | 0.2303 | 0.7697 |  | 0.2171 | 0.7829 |  | 0.7829 | 0.2171 |  | 0.1250 | 0.8750 |  | 0.4671 | 0.5329 |  | 0.2566 | 0.7434 |  | 0.5329 | 0.4671 |  | 0.2303 | 0.7697 |  | 0.7697 | 0.2303 |  | 0.5592 | 0.4408 |  | 0.3618 | 0.6382 | |
| Mexican Amerindian | | 0.3222 | 0.6778 |  | 0.5333 | 0.4667 |  | 0.4333 | 0.5667 |  | 0.2389 | 0.7611 |  | **0.1778** | 0.8222 |  | 0.3444 | 0.6556 |  | 0.8778 | **0.1222** |  | 0.0444 | 0.9556 |  | 0.4278 | 0.5722 |  | 0.3000 | 0.7000 |  | 0.5611 | 0.4389 |  | 0.2889 | 0.7111 |  | 0.8444 | 0.1556 |  | 0.5444 | 0.4556 |  | 0.4944 | 0.5056 | |
| Mexico Mexican | | 0.5373 | 0.4627 |  | 0.6119 | 0.3881 |  | 0.4701 | 0.5299 |  | 0.3657 | 0.6343 |  | 0.2313 | 0.7687 |  | 0.2090 | 0.7910 |  | 0.7612 | 0.2388 |  | 0.3134 | 0.6866 |  | 0.3806 | 0.6194 |  | 0.2463 | 0.7537 |  | 0.6194 | 0.3806 |  | 0.4030 | 0.5970 |  | 0.8955 | **0.1045** |  | 0.5000 | 0.5000 |  | 0.3358 | 0.6642 | |
| Veracruz Mexican | | 0.4554 | 0.5446 |  | 0.5893 | 0.4107 |  | 0.4821 | 0.5179 |  | 0.3929 | 0.6071 |  | 0.2768 | 0.7232 |  | 0.3661 | 0.6339 |  | 0.7232 | 0.2768 |  | 0.2411 | 0.7589 |  | 0.4286 | 0.5714 |  | 0.3036 | 0.6964 |  | 0.4643 | 0.5357 |  | 0.3482 | 0.6518 |  | 0.8036 | 0.1964 |  | 0.5893 | 0.4107 |  | 0.3661 | 0.6339 | |
| Jalisco Mexican | | 0.4300 | 0.5700 |  | 0.7100 | 0.2900 |  | 0.5300 | 0.4700 |  | 0.3200 | 0.6800 |  | 0.3100 | 0.6900 |  | 0.2900 | 0.7100 |  | 0.7000 | 0.3000 |  | 0.2800 | 0.7200 |  | 0.4300 | 0.5700 |  | 0.3400 | 0.6600 |  | 0.5800 | 0.4200 |  | 0.4000 | 0.6000 |  | 0.6900 | 0.3100 |  | 0.5800 | 0.4200 |  | 0.4700 | 0.5300 | |
| Chilhuahua Mexican | | 0.3171 | 0.6829 |  | 0.6829 | 0.3171 |  | 0.5488 | 0.4512 |  | 0.3841 | 0.6159 |  | 0.3537 | 0.6463 |  | 0.2805 | 0.7195 |  | 0.7317 | 0.2683 |  | 0.2927 | 0.7073 |  | 0.4390 | 0.5610 |  | 0.3780 | 0.6220 |  | 0.5549 | 0.4451 |  | 0.3963 | 0.6037 |  | 0.7317 | 0.2683 |  | 0.4024 | 0.5976 |  | 0.4939 | 0.5061 | |
| Uruguayan | | 0.5840 | 0.4160 |  | 0.3969 | 0.6031 |  | 0.4733 | 0.5267 |  | 0.5267 | 0.4733 |  | 0.5305 | 0.4695 |  | 0.5649 | 0.4351 |  | 0.4542 | 0.5458 |  | 0.6794 | 0.3206 |  | 0.5725 | 0.4275 |  | 0.6603 | 0.3397 |  | 0.3855 | 0.6145 |  | 0.4389 | 0.5611 |  | 0.4466 | 0.5534 |  | 0.5458 | 0.4542 |  | 0.5954 | 0.4046 | |
| Dane | | 0.5250 | 0.4750 |  | 0.3000 | 0.7000 |  | 0.4188 | 0.5813 |  | 0.5063 | 0.4938 |  | 0.6813 | 0.3188 |  | 0.6500 | 0.3500 |  | 0.5625 | 0.4375 |  | 0.5813 | 0.4188 |  | 0.5938 | 0.4063 |  | 0.5000 | 0.5000 |  | 0.6063 | 0.3938 |  | 0.4688 | 0.5313 |  | 0.5563 | 0.4438 |  | 0.5188 | 0.4813 |  | 0.4688 | 0.5313 | |
| Central Spanish | | 0.5070 | 0.4930 |  | 0.4014 | 0.5986 |  | 0.4085 | 0.5915 |  | 0.5352 | 0.4648 |  | 0.5563 | 0.4437 |  | 0.7535 | 0.2465 |  | 0.5423 | 0.4577 |  | 0.5986 | 0.4014 |  | 0.6268 | 0.3732 |  | 0.6127 | 0.3873 |  | 0.4014 | 0.5986 |  | 0.4225 | 0.5775 |  | 0.5211 | 0.4789 |  | 0.6056 | 0.3944 |  | 0.4225 | 0.5775 | |
| Hungarian | | 0.5179 | 0.4821 |  | 0.3897 | 0.6103 |  | 0.4333 | 0.5667 |  | 0.4872 | 0.5128 |  | 0.4744 | 0.5256 |  | 0.6846 | 0.3154 |  | 0.5359 | 0.4641 |  | 0.5333 | 0.4667 |  | 0.5821 | 0.4179 |  | 0.5385 | 0.4615 |  | 0.4667 | 0.5333 |  | 0.4487 | 0.5513 |  | 0.4590 | 0.5410 |  | 0.5513 | 0.4487 |  | 0.5769 | 0.4231 | |
| Basque | | 0.5417 | 0.4583 |  | 0.2750 | 0.7250 |  | 0.4333 | 0.5667 |  | 0.5000 | 0.5000 |  | 0.5333 | 0.4667 |  | 0.6167 | 0.3833 |  | 0.6333 | 0.3667 |  | 0.5167 | 0.4833 |  | 0.4667 | 0.5333 |  | 0.6500 | 0.3500 |  | 0.3417 | 0.6583 |  | 0.5167 | 0.4833 |  | 0.5583 | 0.4417 |  | 0.5833 | 0.4167 |  | 0.4000 | 0.6000 | |
| Uygur | | 0.5479 | 0.4521 |  | 0.3085 | 0.6915 |  | 0.5426 | 0.4574 |  | 0.6649 | 0.3351 |  | 0.4043 | 0.5957 |  | 0.6011 | 0.3989 |  | 0.4255 | 0.5745 |  | 0.6862 | 0.3138 |  | 0.6330 | 0.3670 |  | 0.5957 | 0.4043 |  | 0.4681 | 0.5319 |  | 0.7128 | 0.2872 |  | 0.3511 | 0.6489 |  | 0.6064 | 0.3936 |  | 0.4787 | 0.5213 | |
| Kyrgyz | | 0.5492 | 0.4508 |  | 0.2085 | 0.7915 |  | 0.5610 | 0.4390 |  | 0.6610 | 0.3390 |  | 0.5051 | 0.4949 |  | 0.5831 | 0.4169 |  | 0.3932 | 0.6068 |  | 0.7339 | 0.2661 |  | 0.6153 | 0.3847 |  | 0.6186 | 0.3814 |  | 0.5017 | 0.4983 |  | 0.7424 | 0.2576 |  | 0.3492 | 0.6508 |  | 0.7169 | 0.2831 |  | 0.5542 | 0.4458 | |
| Kazakh | | 0.5824 | 0.4176 |  | 0.2253 | 0.7747 |  | 0.5330 | 0.4670 |  | 0.7198 | 0.2802 |  | 0.4451 | 0.5549 |  | 0.5879 | 0.4121 |  | 0.4121 | 0.5879 |  | 0.6923 | 0.3077 |  | 0.5714 | 0.4286 |  | 0.5879 | 0.4121 |  | 0.4615 | 0.5385 |  | 0.6978 | 0.3022 |  | 0.3516 | 0.6484 |  | 0.7033 | 0.2967 |  | 0.5879 | 0.4121 | |
| Beijing Han | | 0.4433 | 0.5567 |  | 0.1392 | 0.8608 |  | 0.7165 | 0.2835 |  | 0.6598 | 0.3402 |  | 0.4072 | 0.5928 |  | 0.5206 | 0.4794 |  | 0.4227 | 0.5773 |  | 0.8454 | 0.1546 |  | 0.7010 | 0.2990 |  | 0.5103 | 0.4897 |  | 0.5361 | 0.4639 |  | 0.8505 | 0.1495 |  | 0.2680 | 0.7320 |  | 0.7165 | 0.2835 |  | 0.6134 | 0.3866 | |
| Tibet Tibetan | | 0.5000 | 0.5000 |  | 0.2439 | 0.7561 |  | 0.5691 | 0.4309 |  | 0.7317 | 0.2683 |  | 0.4675 | 0.5325 |  | 0.6545 | 0.3455 |  | 0.2927 | 0.7073 |  | 0.8780 | 0.1220 |  | 0.5894 | 0.4106 |  | 0.5203 | 0.4797 |  | 0.3699 | 0.6301 |  | 0.8537 | 0.1463 |  | 0.2927 | 0.7073 |  | 0.6707 | 0.3293 |  | 0.5894 | 0.4106 | |
| Qinghai Tibetan | | 0.5410 | 0.4590 |  | 0.2377 | 0.7623 |  | 0.5984 | 0.4016 |  | 0.6475 | 0.3525 |  | 0.4098 | 0.5902 |  | 0.6434 | 0.3566 |  | 0.3156 | 0.6844 |  | 0.8607 | 0.1393 |  | 0.6598 | 0.3402 |  | 0.5697 | 0.4303 |  | 0.4303 | 0.5697 |  | 0.8443 | 0.1557 |  | 0.3525 | 0.6475 |  | 0.6721 | 0.3279 |  | 0.6434 | 0.3566 | |
| Bai | | 0.4800 | 0.5200 |  | 0.1680 | 0.8320 |  | 0.6160 | 0.3840 |  | 0.6080 | 0.3920 |  | 0.3640 | 0.6360 |  | 0.5640 | 0.4360 |  | 0.3840 | 0.6160 |  | 0.8680 | 0.1320 |  | 0.6720 | 0.3280 |  | 0.6440 | 0.3560 |  | 0.4400 | 0.5600 |  | 0.8600 | 0.1400 |  | 0.3800 | 0.6200 |  | 0.7000 | 0.3000 |  | 0.5280 | 0.4720 | |
| Shanghai Han | | 0.4708 | 0.5292 |  | 0.1257 | 0.8743 |  | 0.7159 | 0.2841 |  | 0.6142 | 0.3858 |  | 0.3858 | 0.6142 |  | 0.5204 | 0.4796 |  | 0.3965 | 0.6035 |  | 0.8345 | 0.1655 |  | 0.6832 | 0.3168 |  | 0.5637 | 0.4363 |  | 0.4814 | 0.5186 |  | 0.8230 | 0.1770 |  | 0.3611 | 0.6389 |  | 0.7460 | 0.2540 |  | 0.5150 | 0.4850 | |
| Guangdong Han | | 0.4483 | 0.5517 |  | 0.0950 | 0.9050 |  | 0.7050 | 0.2950 |  | 0.6333 | 0.3667 |  | 0.3883 | 0.6117 |  | 0.4933 | 0.5067 |  | 0.4667 | 0.5333 |  | 0.8400 | 0.1600 |  | 0.7217 | 0.2783 |  | 0.5683 | 0.4317 |  | 0.4317 | 0.5683 |  | 0.7950 | 0.2050 |  | 0.3600 | 0.6400 |  | 0.7983 | 0.2017 |  | 0.5067 | 0.4933 | |
| She | | 0.3950 | 0.6050 |  | 0.0966 | 0.9034 |  | 0.7143 | 0.2857 |  | 0.6597 | 0.3403 |  | 0.3782 | 0.6218 |  | 0.5420 | 0.4580 |  | 0.3950 | 0.6050 |  | 0.8655 | 0.1345 |  | 0.7857 | 0.2143 |  | 0.4958 | 0.5042 |  | 0.4832 | 0.5168 |  | 0.7941 | 0.2059 |  | 0.2815 | 0.7185 |  | 0.8193 | **0.1807** |  | 0.5714 | 0.4286 | |
| South Korean | | 0.4330 | 0.5670 |  | 0.1273 | 0.8727 |  | 0.6260 | 0.3740 |  | 0.5885 | 0.4115 |  | 0.3780 | 0.6220 |  | 0.5295 | 0.4705 |  | 0.3351 | 0.6649 |  | 0.8552 | 0.1448 |  | 0.6823 | 0.3177 |  | 0.6327 | 0.3673 |  | 0.4625 | 0.5375 |  | 0.8740 | 0.1260 |  | 0.3995 | 0.6005 |  | 0.7560 | 0.2440 |  | 0.5161 | 0.4839 | |
| Xibe | | 0.5426 | 0.4574 |  | 0.1525 | 0.8475 |  | 0.6233 | 0.3767 |  | 0.6323 | 0.3677 |  | 0.4081 | 0.5919 |  | 0.5987 | 0.4013 |  | 0.3004 | 0.6996 |  | 0.7646 | 0.2354 |  | 0.7152 | 0.2848 |  | 0.6166 | 0.3834 |  | 0.5157 | 0.4843 |  | 0.8430 | 0.1570 |  | 0.4058 | 0.5942 |  | 0.8027 | 0.1973 |  | 0.5785 | 0.4215 | |
| Yi | | 0.3770 | 0.6230 |  | **0.0943** | 0.9057 |  | 0.5697 | 0.4303 |  | 0.6557 | 0.3443 |  | 0.3689 | 0.6311 |  | 0.4221 | 0.5779 |  | 0.3770 | 0.6230 |  | 0.8975 | 0.1025 |  | 0.7049 | 0.2951 |  | 0.5164 | 0.4836 |  | 0.3197 | 0.6803 |  | 0.8852 | **0.1148** |  | 0.5082 | 0.4918 |  | 0.7910 | 0.2090 |  | 0.5738 | 0.4262 | |
| Tujia | | 0.4682 | 0.5318 |  | 0.1165 | 0.8835 |  | 0.6737 | 0.3263 |  | 0.6525 | 0.3475 |  | 0.4428 | 0.5572 |  | 0.5508 | 0.4492 |  | 0.4025 | 0.5975 |  | 0.8602 | 0.1398 |  | 0.7542 | 0.2458 |  | 0.5636 | 0.4364 |  | 0.4640 | 0.5360 |  | 0.8305 | 0.1695 |  | 0.3814 | 0.6186 |  | 0.7606 | 0.2394 |  | 0.5530 | 0.4470 | |

Continued

| Populations | HLD92 | |  | HLD93 | |  | HLD97 | |  | HLD99 | |  | HLD101 | |  | HLD111 | |  | HLD114 | |  | HLD118 | |  | HLD122 | |  | HLD124 | |  | HLD125 | |  | HLD128 | |  | HLD131 | |  | HLD133 | |  | HLD136 | |
| --- | --- | --- | --- | --- | --- | --- | --- | --- | --- | --- | --- | --- | --- | --- | --- | --- | --- | --- | --- | --- | --- | --- | --- | --- | --- | --- | --- | --- | --- | --- | --- | --- | --- | --- | --- | --- | --- | --- | --- | --- | --- | --- | --- | --- |
|  | DIP+ | DIP- |  | DIP+ | DIP- |  | DIP+ | DIP- |  | DIP+ | DIP- |  | DIP+ | DIP- |  | DIP+ | DIP- |  | DIP+ | DIP- |  | DIP+ | DIP- |  | DIP+ | DIP- |  | DIP+ | DIP- |  | DIP+ | DIP- |  | DIP+ | DIP- |  | DIP+ | DIP- |  | DIP+ | DIP- |  | DIP+ | DIP- |
| Yucatan Mexican | 0.4408 | 0.5592 |  | 0.4145 | 0.5855 |  | 0.5724 | 0.4276 |  | 0.5724 | 0.4276 |  | 0.5461 | 0.4539 |  | 0.6316 | 0.3684 |  | 0.6118 | 0.3882 |  | 0.7368 | 0.2632 |  | 0.7697 | 0.2303 |  | 0.3816 | 0.6184 |  | 0.7697 | 0.2303 |  | 0.6908 | 0.3092 |  | 0.5329 | 0.4671 |  | 0.5855 | 0.4145 |  | 0.5395 | 0.4605 |
| Mexican Amerindian | 0.5333 | 0.4667 |  | 0.3667 | 0.6333 |  | 0.6389 | 0.3611 |  | 0.6111 | 0.3889 |  | 0.5833 | 0.4167 |  | 0.8000 | 0.2000 |  | 0.5278 | 0.4722 |  | 0.8444 | 0.1556 |  | 0.7389 | 0.2611 |  | 0.3778 | 0.6222 |  | 0.9167 | **0.0833** |  | 0.6833 | 0.3167 |  | 0.6111 | 0.3889 |  | 0.6167 | 0.3833 |  | 0.3000 | 0.7000 |
| Mexico Mexican | 0.5672 | 0.4328 |  | 0.4776 | 0.5224 |  | 0.5224 | 0.4776 |  | 0.5149 | 0.4851 |  | 0.4104 | 0.5896 |  | 0.6493 | 0.3507 |  | 0.5522 | 0.4478 |  | 0.7463 | 0.2537 |  | 0.6642 | 0.3358 |  | 0.4552 | 0.5448 |  | 0.7836 | 0.2164 |  | 0.5970 | 0.4030 |  | 0.6194 | 0.3806 |  | 0.6269 | 0.3731 |  | 0.4552 | 0.5448 |
| Veracruz Mexican | 0.5536 | 0.4464 |  | 0.3571 | 0.6429 |  | 0.5446 | 0.4554 |  | 0.6875 | 0.3125 |  | 0.5000 | 0.5000 |  | 0.6786 | 0.3214 |  | 0.5982 | 0.4018 |  | 0.6786 | 0.3214 |  | 0.6607 | 0.3393 |  | 0.4286 | 0.5714 |  | 0.8036 | 0.1964 |  | 0.5893 | 0.4107 |  | 0.6429 | 0.3571 |  | 0.5714 | 0.4286 |  | 0.4286 | 0.5714 |
| Jalisco Mexican | 0.5800 | 0.4200 |  | 0.4200 | 0.5800 |  | 0.5900 | 0.4100 |  | 0.6200 | 0.3800 |  | 0.5700 | 0.4300 |  | 0.6100 | 0.3900 |  | 0.5300 | 0.4700 |  | 0.6800 | 0.3200 |  | 0.6400 | 0.3600 |  | 0.4600 | 0.5400 |  | 0.6900 | 0.3100 |  | 0.5600 | 0.4400 |  | 0.5400 | 0.4600 |  | 0.5500 | 0.4500 |  | 0.3700 | 0.6300 |
| Chilhuahua Mexican | 0.5427 | 0.4573 |  | 0.4451 | 0.5549 |  | 0.5854 | 0.4146 |  | 0.5244 | 0.4756 |  | 0.5915 | 0.4085 |  | 0.5671 | 0.4329 |  | 0.5488 | 0.4512 |  | 0.7378 | 0.2622 |  | 0.7073 | 0.2927 |  | 0.4390 | 0.5610 |  | 0.7622 | 0.2378 |  | 0.5305 | 0.4695 |  | 0.4512 | 0.5488 |  | 0.5488 | 0.4512 |  | 0.4329 | 0.5671 |
| Uruguayan | 0.4122 | 0.5878 |  | 0.5802 | 0.4198 |  | 0.5153 | 0.4847 |  | 0.6221 | 0.3779 |  | 0.5153 | 0.4847 |  | 0.5115 | 0.4885 |  | 0.4084 | 0.5916 |  | 0.3931 | 0.6069 |  | 0.4313 | 0.5687 |  | 0.6412 | 0.3588 |  | 0.4580 | 0.5420 |  | 0.4466 | 0.5534 |  | 0.5267 | 0.4733 |  | 0.5420 | 0.4580 |  | 0.5611 | 0.4389 |
| Dane | 0.4688 | 0.5313 |  | 0.6063 | 0.3938 |  | 0.5313 | 0.4688 |  | 0.5688 | 0.4313 |  | 0.3688 | 0.6313 |  | 0.5375 | 0.4625 |  | 0.3688 | 0.6313 |  | 0.4563 | 0.5438 |  | 0.3813 | 0.6188 |  | 0.5563 | 0.4438 |  | 0.5438 | 0.4563 |  | 0.4313 | 0.5688 |  | 0.5250 | 0.4750 |  | 0.6063 | 0.3938 |  | 0.4938 | 0.5063 |
| Central Spanish | 0.4859 | 0.5141 |  | 0.5070 | 0.4930 |  | 0.5211 | 0.4789 |  | 0.5986 | 0.4014 |  | 0.5141 | 0.4859 |  | 0.5000 | 0.5000 |  | 0.4859 | 0.5141 |  | 0.4155 | 0.5845 |  | 0.4014 | 0.5986 |  | 0.6479 | 0.3521 |  | 0.4648 | 0.5352 |  | 0.4507 | 0.5493 |  | 0.5000 | 0.5000 |  | 0.6268 | 0.3732 |  | 0.5563 | 0.4437 |
| Hungarian | 0.4154 | 0.5846 |  | 0.5205 | 0.4795 |  | 0.4974 | 0.5026 |  | 0.5667 | 0.4333 |  | 0.4821 | 0.5179 |  | 0.5872 | 0.4128 |  | 0.3462 | 0.6538 |  | 0.4744 | 0.5256 |  | 0.4923 | 0.5077 |  | 0.6385 | 0.3615 |  | 0.5359 | 0.4641 |  | 0.4846 | 0.5154 |  | 0.5795 | 0.4205 |  | 0.5744 | 0.4256 |  | 0.4821 | 0.5179 |
| Basque | 0.4167 | 0.5833 |  | 0.4833 | 0.5167 |  | 0.6083 | 0.3917 |  | 0.6333 | 0.3667 |  | 0.4333 | 0.5667 |  | 0.5583 | 0.4417 |  | 0.3250 | 0.6750 |  | 0.4250 | 0.5750 |  | 0.3333 | 0.6667 |  | 0.6833 | 0.3167 |  | 0.4750 | 0.5250 |  | 0.5083 | 0.4917 |  | 0.3833 | 0.6167 |  | 0.6167 | 0.3833 |  | 0.7000 | 0.3000 |
| Uygur | 0.5213 | 0.4787 |  | 0.4787 | 0.5213 |  | 0.3989 | 0.6011 |  | 0.6170 | 0.3830 |  | 0.5957 | 0.4043 |  | 0.3032 | 0.6968 |  | 0.3883 | 0.6117 |  | 0.5798 | 0.4202 |  | 0.3670 | 0.6330 |  | 0.5160 | 0.4840 |  | 0.5160 | 0.4840 |  | 0.4309 | 0.5691 |  | 0.4947 | 0.5053 |  | 0.4202 | 0.5798 |  | 0.5160 | 0.4840 |
| Kyrgyz | 0.5559 | 0.4441 |  | 0.5542 | 0.4458 |  | 0.3729 | 0.6271 |  | 0.7356 | 0.2644 |  | 0.5068 | 0.4932 |  | 0.2593 | 0.7407 |  | 0.4102 | 0.5898 |  | 0.7356 | 0.2644 |  | 0.3373 | 0.6627 |  | 0.6525 | 0.3475 |  | 0.4983 | 0.5017 |  | 0.3797 | 0.6203 |  | 0.4542 | 0.5458 |  | 0.4441 | 0.5559 |  | 0.4627 | 0.5373 |
| Kazakh | 0.5549 | 0.4451 |  | 0.5934 | 0.4066 |  | 0.3626 | 0.6374 |  | 0.6099 | 0.3901 |  | 0.5440 | 0.4560 |  | 0.2912 | 0.7088 |  | 0.4286 | 0.5714 |  | 0.6538 | 0.3462 |  | 0.3626 | 0.6374 |  | 0.5824 | 0.4176 |  | 0.4670 | 0.5330 |  | 0.4066 | 0.5934 |  | 0.4505 | 0.5495 |  | 0.4505 | 0.5495 |  | 0.4890 | 0.5110 |
| Beijing Han | 0.4639 | 0.5361 |  | 0.5464 | 0.4536 |  | 0.3763 | 0.6237 |  | 0.7732 | 0.2268 |  | 0.4897 | 0.5103 |  | 0.0876 | 0.9124 |  | 0.2887 | 0.7113 |  | 0.9330 | 0.0670 |  | 0.2526 | 0.7474 |  | 0.5619 | 0.4381 |  | 0.4485 | 0.5515 |  | 0.3247 | 0.6753 |  | 0.3763 | 0.6237 |  | 0.3763 | 0.6237 |  | 0.5670 | 0.4330 |
| Tibet Tibetan | 0.5122 | 0.4878 |  | 0.5854 | 0.4146 |  | 0.2764 | 0.7236 |  | 0.7805 | 0.2195 |  | 0.4797 | 0.5203 |  | 0.0854 | 0.9146 |  | 0.2602 | 0.7398 |  | 0.9187 | 0.0813 |  | 0.3293 | 0.6707 |  | 0.5528 | 0.4472 |  | 0.4390 | 0.5610 |  | 0.2927 | 0.7073 |  | 0.4512 | 0.5488 |  | 0.4350 | 0.5650 |  | 0.5772 | 0.4228 |
| Qinghai Tibetan | 0.4344 | 0.5656 |  | 0.5984 | 0.4016 |  | 0.3238 | 0.6762 |  | 0.8361 | 0.1639 |  | 0.4877 | 0.5123 |  | 0.1025 | 0.8975 |  | 0.2787 | 0.7213 |  | 0.9180 | 0.0820 |  | 0.2951 | 0.7049 |  | 0.4385 | 0.5615 |  | 0.4180 | 0.5820 |  | 0.3074 | 0.6926 |  | 0.3975 | 0.6025 |  | 0.3893 | 0.6107 |  | 0.5533 | 0.4467 |
| Bai | 0.4640 | 0.5360 |  | 0.5400 | 0.4600 |  | 0.3160 | 0.6840 |  | 0.8000 | 0.2000 |  | 0.4480 | 0.5520 |  | 0.1120 | 0.8880 |  | **0.1840** | 0.8160 |  | 0.8920 | 0.1080 |  | 0.2400 | 0.7600 |  | 0.5360 | 0.4640 |  | 0.4520 | 0.5480 |  | 0.2520 | 0.7480 |  | 0.4280 | 0.5720 |  | 0.3880 | 0.6120 |  | 0.5120 | 0.4880 |
| Shanghai Han | 0.4434 | 0.5566 |  | 0.5469 | 0.4531 |  | 0.3372 | 0.6628 |  | 0.8699 | 0.1301 |  | 0.4681 | 0.5319 |  | 0.0841 | 0.9159 |  | 0.2717 | 0.7283 |  | 0.9372 | 0.0628 |  | 0.2389 | 0.7611 |  | 0.5416 | 0.4584 |  | 0.3575 | 0.6425 |  | 0.3159 | 0.6841 |  | 0.3469 | 0.6531 |  | 0.3602 | 0.6398 |  | 0.5230 | 0.4770 |
| Guangdong Han | 0.5000 | 0.5000 |  | 0.5600 | 0.4400 |  | 0.3617 | 0.6383 |  | 0.8850 | 0.1150 |  | 0.4733 | 0.5267 |  | 0.1050 | 0.8950 |  | 0.2450 | 0.7550 |  | 0.9200 | 0.0800 |  | 0.1983 | 0.8017 |  | 0.5117 | 0.4883 |  | 0.3817 | 0.6183 |  | 0.3417 | 0.6583 |  | 0.3367 | 0.6633 |  | 0.3500 | 0.6500 |  | 0.4717 | 0.5283 |
| She | 0.4454 | 0.5546 |  | 0.6387 | 0.3613 |  | 0.3697 | 0.6303 |  | 0.8866 | 0.1134 |  | 0.3824 | 0.6176 |  | 0.1261 | 0.8739 |  | 0.2563 | 0.7437 |  | 0.8613 | 0.1387 |  | **0.1429** | 0.8571 |  | 0.5126 | 0.4874 |  | 0.3992 | 0.6008 |  | 0.3109 | 0.6891 |  | 0.3445 | 0.6555 |  | 0.3109 | 0.6891 |  | 0.5882 | 0.4118 |
| South Korean | 0.4196 | 0.5804 |  | 0.6072 | 0.3928 |  | 0.3298 | 0.6702 |  | 0.9142 | **0.0858** |  | 0.4330 | 0.5670 |  | **0.0724** | 0.9276 |  | 0.2855 | 0.7145 |  | 0.9504 | 0.0496 |  | 0.2641 | 0.7359 |  | 0.5563 | 0.4437 |  | 0.3552 | 0.6448 |  | 0.3472 | 0.6528 |  | 0.3619 | 0.6381 |  | 0.3767 | 0.6233 |  | 0.5818 | 0.4182 |
| Xibe | 0.4821 | 0.5179 |  | 0.6211 | 0.3789 |  | 0.3744 | 0.6256 |  | 0.8430 | 0.1570 |  | 0.4888 | 0.5112 |  | 0.1233 | 0.8767 |  | 0.3206 | 0.6794 |  | 0.9148 | 0.0852 |  | 0.2735 | 0.7265 |  | 0.5874 | 0.4126 |  | 0.4081 | 0.5919 |  | 0.3610 | 0.6390 |  | 0.3139 | 0.6861 |  | 0.4058 | 0.5942 |  | 0.5426 | 0.4574 |
| Yi | 0.4180 | 0.5820 |  | 0.6926 | 0.3074 |  | 0.3156 | 0.6844 |  | 0.8402 | 0.1598 |  | 0.4959 | 0.5041 |  | 0.0779 | 0.9221 |  | 0.2459 | 0.7541 |  | 0.8893 | 0.1107 |  | 0.2459 | 0.7541 |  | 0.5943 | 0.4057 |  | 0.4262 | 0.5738 |  | **0.1475** | 0.8525 |  | 0.2869 | 0.7131 |  | 0.3238 | 0.6762 |  | 0.3525 | 0.6475 |
| Tujia | 0.4555 | 0.5445 |  | 0.5593 | 0.4407 |  | 0.3390 | 0.6610 |  | 0.8644 | 0.1356 |  | 0.4661 | 0.5339 |  | 0.0911 | 0.9089 |  | 0.2352 | 0.7648 |  | 0.9555 | 0.0445 |  | 0.2331 | 0.7669 |  | 0.5826 | 0.4174 |  | 0.3517 | 0.6483 |  | 0.3496 | 0.6504 |  | 0.3347 | 0.6653 |  | 0.3623 | 0.6377 |  | 0.5275 | 0.4725 |
| Values of MAF (0.0700-0.2000) were shown in bold | | | | | | | | | | | | | | | | | | | | | | | | | | | | | | | | | | | | | | | | | | | | |

| Supplementary Table 2. *Fst* and *p*-values of pairwise DIP loci between Kyrgyz group and other groups at 30 DIP loci | | | | | | | | | | | | | | | | | | | | | | | | | | | | | | | | | | | | | | | | | | | | | | | | |
| --- | --- | --- | --- | --- | --- | --- | --- | --- | --- | --- | --- | --- | --- | --- | --- | --- | --- | --- | --- | --- | --- | --- | --- | --- | --- | --- | --- | --- | --- | --- | --- | --- | --- | --- | --- | --- | --- | --- | --- | --- | --- | --- | --- | --- | --- | --- | --- | --- |
| **Loci** | **Yucatan Mexican** | | **Mexican Amerindian** | | **Mexico Mexican** | | **Veracruz Mexican** | | **Jalisco Mexican** | | **Chilhuahua Mexican** | | **Uruguayan** | | **Dane** | | **Central Spanish** | | **Hungarian** | | **Basque** | | **Uygur** | | **Kazakh** | | **Beijing Han** | | **Tibet Tibetan** | | **Qinghai Tibetan** | | **Bai** | | **Shanghai Han** | | **Guangdong** Han | | **She** | | **South Korean** | | **Xibe** | | **Yi** | | **Tujia** | |
|  | ***Fst*** | ***p*** | ***Fst*** | ***p*** | ***Fst*** | ***p*** | ***Fst*** | ***p*** | ***Fst*** | ***p*** | ***Fst*** | ***p*** | ***Fst*** | ***p*** | ***Fst*** | ***p*** | ***Fst*** | ***p*** | ***Fst*** | ***p*** | ***Fst*** | ***p*** | ***Fst*** | ***p*** | ***Fst*** | ***p*** | ***Fst*** | ***p*** | ***Fst*** | ***p*** | ***Fst*** | ***p*** | ***Fst*** | ***p*** | ***Fst*** | ***p*** | ***Fst*** | ***p*** | ***Fst*** | ***p*** | ***Fst*** | ***p*** | ***Fst*** | ***p*** | ***Fst*** | ***p*** | ***Fst*** | ***p*** |
| HLD6 | 0.0219 | 0.0176 | 0.0934 | 0.0000 | -0.0043 | 1.0000 | 0.0123 | 0.0978 | 0.0222 | 0.0332 | 0.0973 | 0.0000 | -0.0003 | 0.5591 | -0.0028 | 1.0000 | -0.0008 | 0.5807 | -0.0004 | 0.5660 | -0.0050 | 1.0000 | -0.0035 | 1.0000 | -0.0015 | 0.7390 | 0.0190 | 0.0088 | 0.0018 | 0.2991 | -0.0028 | 1.0000 | 0.0066 | 0.0899 | 0.0109 | 0.0029 | 0.0185 | 0.0000 | 0.0435 | 0.0000 | 0.0252 | 0.0000 | -0.0019 | 1.0000 | 0.0545 | 0.0000 | 0.0111 | 0.0147 |
| HLD39 | 0.2646 | 0.0000 | 0.2197 | 0.0000 | 0.3105 | 0.0000 | 0.2871 | 0.0000 | 0.4210 | 0.0000 | 0.3876 | 0.0000 | 0.0839 | 0.0000 | 0.0194 | 0.0264 | 0.0899 | 0.0000 | 0.0762 | 0.0000 | 0.0079 | 0.1466 | 0.0240 | 0.0068 | -0.0029 | 1.0000 | 0.0120 | 0.0508 | 0.0007 | 0.4330 | -0.0003 | 0.5288 | 0.0024 | 0.2698 | 0.0247 | 0.0000 | 0.0473 | 0.0000 | 0.0391 | 0.0010 | 0.0223 | 0.0000 | 0.0084 | 0.0303 | 0.0412 | 0.0000 | 0.0280 | 0.0000 |
| HLD40 | 0.0239 | 0.0186 | 0.0283 | 0.0020 | 0.0115 | 0.0929 | 0.0065 | 0.2180 | -0.0047 | 1.0000 | -0.0040 | 1.0000 | 0.0125 | 0.0225 | 0.0353 | 0.0020 | 0.0406 | 0.0029 | 0.0298 | 0.0000 | 0.0267 | 0.0156 | -0.0032 | 1.0000 | -0.0024 | 1.0000 | 0.0452 | 0.0000 | -0.0029 | 1.0000 | -0.0003 | 0.5435 | 0.0030 | 0.2571 | 0.0509 | 0.0000 | 0.0420 | 0.0000 | 0.0447 | 0.0000 | 0.0072 | 0.0196 | 0.0059 | 0.0616 | -0.0030 | 1.0000 | 0.0243 | 0.0020 |
| HLD45 | 0.1817 | 0.0000 | 0.2913 | 0.0000 | 0.1584 | 0.0000 | 0.1327 | 0.0000 | 0.2023 | 0.0000 | 0.1413 | 0.0000 | 0.0348 | 0.0000 | 0.0459 | 0.0000 | 0.0291 | 0.0098 | 0.0586 | 0.0000 | 0.0491 | 0.0010 | -0.0036 | 1.0000 | 0.0043 | 0.1603 | -0.0036 | 1.0000 | 0.0085 | 0.0577 | -0.0026 | 1.0000 | 0.0033 | 0.2072 | 0.0034 | 0.0763 | 0.0000 | 0.4673 | -0.0029 | 1.0000 | 0.0097 | 0.0117 | -0.0002 | 0.5376 | -0.0030 | 1.0000 | -0.0017 | 1.0000 |
| HLD48 | 0.1351 | 0.0000 | 0.1892 | 0.0000 | 0.1332 | 0.0000 | 0.0931 | 0.0000 | 0.0673 | 0.0000 | 0.0411 | 0.0000 | -0.0014 | 1.0000 | 0.0565 | 0.0000 | 0.0012 | 0.3832 | -0.0001 | 0.4858 | -0.0033 | 1.0000 | 0.0169 | 0.0176 | 0.0039 | 0.2229 | 0.0159 | 0.0156 | 0.0001 | 0.4115 | 0.0152 | 0.0205 | 0.0365 | 0.0000 | 0.0274 | 0.0000 | 0.0256 | 0.0000 | 0.0289 | 0.0000 | 0.0309 | 0.0000 | 0.0169 | 0.0020 | 0.0338 | 0.0000 | 0.0060 | 0.0401 |
| HLD56 | 0.2237 | 0.0000 | 0.1031 | 0.0000 | 0.2309 | 0.0000 | 0.0839 | 0.0000 | 0.1481 | 0.0000 | 0.1602 | 0.0000 | -0.0022 | 1.0000 | 0.0051 | 0.2317 | 0.0546 | 0.0020 | 0.0196 | 0.0039 | -0.0030 | 1.0000 | -0.0031 | 1.0000 | -0.0041 | 1.0000 | 0.0044 | 0.1808 | 0.0075 | 0.0948 | 0.0045 | 0.1945 | -0.0025 | 1.0000 | 0.0066 | 0.0215 | 0.0143 | 0.0020 | 0.0003 | 0.4115 | 0.0043 | 0.0665 | -0.0016 | 1.0000 | 0.0477 | 0.0000 | 0.0002 | 0.4624 |
| HLD58 | 0.2497 | 0.0000 | 0.3587 | 0.0000 | 0.2254 | 0.0000 | 0.1857 | 0.0000 | 0.1623 | 0.0000 | 0.1968 | 0.0000 | 0.0049 | 0.1251 | 0.0524 | 0.0000 | 0.0399 | 0.0020 | 0.0382 | 0.0000 | 0.1036 | 0.0000 | -0.0015 | 0.7449 | -0.0031 | 1.0000 | -0.0019 | 1.0000 | 0.0186 | 0.0127 | 0.0098 | 0.0616 | -0.0028 | 1.0000 | -0.0013 | 1.0000 | 0.0092 | 0.0215 | -0.0033 | 1.0000 | 0.0057 | 0.0508 | 0.0166 | 0.0039 | -0.0025 | 1.0000 | -0.0018 | 1.0000 |
| HLD64 | 0.5089 | 0.0000 | 0.5971 | 0.0000 | 0.3046 | 0.0000 | 0.3831 | 0.0000 | 0.3411 | 0.0000 | 0.3274 | 0.0000 | 0.0045 | 0.1545 | 0.0498 | 0.0000 | 0.0386 | 0.0010 | 0.0830 | 0.0000 | 0.0992 | 0.0000 | 0.0021 | 0.3118 | 0.0008 | 0.4164 | 0.0301 | 0.0010 | 0.0551 | 0.0000 | 0.0414 | 0.0000 | 0.0471 | 0.0000 | 0.0299 | 0.0000 | 0.0314 | 0.0000 | 0.0451 | 0.0000 | 0.0438 | 0.0000 | 0.0004 | 0.4135 | 0.0723 | 0.0000 | 0.0451 | 0.0000 |
| HLD67 | 0.0397 | 0.0020 | 0.0651 | 0.0000 | 0.1002 | 0.0000 | 0.0632 | 0.0000 | 0.0620 | 0.0000 | 0.0572 | 0.0000 | 0.0009 | 0.3949 | -0.0033 | 1.0000 | -0.0043 | 1.0000 | -0.0001 | 0.4526 | 0.0392 | 0.0059 | -0.0032 | 1.0000 | 0.0004 | 0.4516 | 0.0124 | 0.0362 | -0.0015 | 1.0000 | 0.0012 | 0.3441 | 0.0039 | 0.1945 | 0.0089 | 0.0088 | 0.0236 | 0.0010 | 0.0599 | 0.0000 | 0.0083 | 0.0176 | 0.0200 | 0.0010 | 0.0143 | 0.0127 | 0.0412 | 0.0000 |
| HLD70 | 0.2214 | 0.0000 | 0.1780 | 0.0000 | 0.2311 | 0.0000 | 0.1723 | 0.0000 | 0.1375 | 0.0000 | 0.1060 | 0.0000 | 0.0009 | 0.3754 | 0.0248 | 0.0117 | -0.0044 | 1.0000 | 0.0111 | 0.0176 | -0.0029 | 1.0000 | -0.0025 | 1.0000 | -0.0018 | 0.8720 | 0.0207 | 0.0078 | 0.0170 | 0.0078 | 0.0021 | 0.3148 | -0.0015 | 1.0000 | 0.0049 | 0.0420 | 0.0036 | 0.1154 | 0.0276 | 0.0029 | -0.0010 | 1.0000 | -0.0020 | 1.0000 | 0.0183 | 0.0078 | 0.0044 | 0.0978 |
| HLD77 | -0.0023 | 1.0000 | 0.0033 | 0.2522 | 0.0227 | 0.0147 | -0.0023 | 0.8172 | 0.0062 | 0.1779 | 0.0016 | 0.3470 | 0.0239 | 0.0049 | 0.0176 | 0.0313 | 0.0154 | 0.0381 | 0.0002 | 0.4448 | 0.0447 | 0.0020 | -0.0012 | 0.6540 | -0.0005 | 0.5181 | -0.0011 | 0.6706 | 0.0312 | 0.0000 | 0.0071 | 0.0772 | 0.0047 | 0.1720 | -0.0005 | 0.7302 | 0.0081 | 0.0215 | -0.0023 | 1.0000 | 0.0015 | 0.2151 | -0.0016 | 1.0000 | 0.0616 | 0.0000 | 0.0009 | 0.3157 |
| HLD81 | 0.4083 | 0.0000 | 0.3438 | 0.0000 | 0.2199 | 0.0000 | 0.2797 | 0.0000 | 0.2243 | 0.0000 | 0.2262 | 0.0000 | 0.1789 | 0.0000 | 0.1522 | 0.0000 | 0.1996 | 0.0000 | 0.1662 | 0.0000 | 0.1082 | 0.0000 | -0.0010 | 0.7019 | 0.0015 | 0.3402 | 0.0290 | 0.0010 | 0.0322 | 0.0010 | 0.0263 | 0.0010 | 0.0363 | 0.0000 | 0.0185 | 0.0000 | 0.0061 | 0.0430 | 0.0045 | 0.1466 | 0.0546 | 0.0000 | 0.0277 | 0.0000 | 0.0557 | 0.0000 | 0.0207 | 0.0000 |
| HLD83 | 0.2872 | 0.0000 | 0.3727 | 0.0000 | 0.4224 | 0.0000 | 0.3205 | 0.0000 | 0.2014 | 0.0000 | 0.2468 | 0.0000 | 0.0175 | 0.0029 | 0.0816 | 0.0000 | 0.0563 | 0.0000 | 0.0230 | 0.0000 | 0.0828 | 0.0000 | -0.0032 | 1.0000 | -0.0034 | 1.0000 | 0.0119 | 0.0244 | 0.0046 | 0.1163 | -0.0027 | 1.0000 | -0.0004 | 0.5386 | -0.0009 | 1.0000 | -0.0014 | 1.0000 | 0.0077 | 0.0518 | 0.0039 | 0.0860 | 0.0050 | 0.0675 | 0.0488 | 0.0000 | 0.0005 | 0.3431 |
| HLD84 | 0.0515 | 0.0000 | 0.0619 | 0.0000 | 0.0958 | 0.0000 | 0.0324 | 0.0108 | 0.0375 | 0.0078 | 0.1867 | 0.0000 | 0.0607 | 0.0000 | 0.0808 | 0.0000 | 0.0243 | 0.0205 | 0.0565 | 0.0000 | 0.0360 | 0.0029 | 0.0246 | 0.0147 | -0.0033 | 1.0000 | -0.0036 | 1.0000 | 0.0023 | 0.2561 | 0.0019 | 0.2639 | -0.0023 | 1.0000 | 0.0009 | 0.2620 | 0.0161 | 0.0049 | 0.0242 | 0.0039 | 0.0024 | 0.1593 | 0.0175 | 0.0020 | 0.0111 | 0.0391 | 0.0029 | 0.1662 |
| HLD88 | 0.0669 | 0.0000 | 0.0036 | 0.2454 | 0.0854 | 0.0000 | 0.0628 | 0.0000 | 0.0083 | 0.1642 | 0.0036 | 0.2581 | 0.0008 | 0.3627 | 0.0106 | 0.0645 | 0.0299 | 0.0068 | -0.0011 | 1.0000 | 0.0415 | 0.0039 | 0.0081 | 0.0919 | -0.0011 | 0.6442 | 0.0039 | 0.2102 | -0.0003 | 0.5435 | 0.0133 | 0.0166 | -0.0014 | 1.0000 | 0.0019 | 0.1662 | 0.0029 | 0.1300 | -0.0023 | 1.0000 | 0.0014 | 0.2307 | -0.0008 | 0.7507 | -0.0021 | 1.0000 | -0.0020 | 1.0000 |
| HLD92 | 0.0224 | 0.0108 | -0.0024 | 1.0000 | -0.0041 | 1.0000 | -0.0052 | 1.0000 | -0.0044 | 1.0000 | -0.0036 | 1.0000 | 0.0377 | 0.0010 | 0.0114 | 0.0567 | 0.0056 | 0.1486 | 0.0367 | 0.0000 | 0.0331 | 0.0088 | -0.0010 | 0.6872 | -0.0036 | 1.0000 | 0.0135 | 0.0352 | 0.0011 | 0.3900 | 0.0261 | 0.0029 | 0.0140 | 0.0225 | 0.0238 | 0.0000 | 0.0047 | 0.0899 | 0.0213 | 0.0078 | 0.0351 | 0.0000 | 0.0090 | 0.0264 | 0.0345 | 0.0000 | 0.0182 | 0.0010 |
| HLD93 | 0.0344 | 0.0020 | 0.0640 | 0.0000 | 0.0071 | 0.1613 | 0.0688 | 0.0000 | 0.0296 | 0.0137 | 0.0198 | 0.0196 | -0.0015 | 1.0000 | 0.0016 | 0.3578 | 0.0002 | 0.4604 | 0.0001 | 0.4633 | 0.0050 | 0.2190 | 0.0078 | 0.0978 | -0.0004 | 0.5142 | -0.0031 | 1.0000 | -0.0009 | 0.6207 | 0.0010 | 0.3294 | -0.0026 | 1.0000 | -0.0012 | 1.0000 | -0.0017 | 1.0000 | 0.0116 | 0.0313 | 0.0043 | 0.0860 | 0.0072 | 0.0264 | 0.0361 | 0.0000 | -0.0019 | 1.0000 |
| HLD97 | 0.0740 | 0.0000 | 0.1287 | 0.0000 | 0.0408 | 0.0010 | 0.0541 | 0.0020 | 0.0862 | 0.0000 | 0.0838 | 0.0000 | 0.0380 | 0.0000 | 0.0464 | 0.0010 | 0.0402 | 0.0039 | 0.0291 | 0.0000 | 0.1011 | 0.0000 | -0.0021 | 1.0000 | -0.0033 | 1.0000 | -0.0033 | 1.0000 | 0.0176 | 0.0117 | 0.0023 | 0.2688 | 0.0041 | 0.1887 | 0.0014 | 0.2160 | -0.0014 | 1.0000 | -0.0032 | 1.0000 | 0.0025 | 0.1378 | -0.0021 | 1.0000 | 0.0042 | 0.1965 | 0.0006 | 0.3705 |
| HLD99 | 0.0573 | 0.0000 | 0.0331 | 0.0020 | 0.1024 | 0.0000 | 0.0008 | 0.4546 | 0.0265 | 0.0186 | 0.0943 | 0.0000 | 0.0275 | 0.0020 | 0.0598 | 0.0000 | 0.0401 | 0.0020 | 0.0603 | 0.0000 | 0.0206 | 0.0342 | 0.0304 | 0.0000 | 0.0341 | 0.0000 | 0.0005 | 0.4477 | 0.0026 | 0.2463 | 0.0248 | 0.0020 | 0.0083 | 0.0547 | 0.0589 | 0.0000 | 0.0687 | 0.0000 | 0.0610 | 0.0000 | 0.1082 | 0.0000 | 0.0313 | 0.0000 | 0.0272 | 0.0000 | 0.0474 | 0.0000 |
| HLD101 | -0.0011 | 0.6461 | 0.0081 | 0.1046 | 0.0140 | 0.0518 | -0.0051 | 1.0000 | 0.0021 | 0.3861 | 0.0104 | 0.0831 | -0.0026 | 1.0000 | 0.0334 | 0.0029 | -0.0041 | 1.0000 | -0.0009 | 0.7439 | 0.0061 | 0.1633 | 0.0124 | 0.0489 | -0.0007 | 0.5269 | -0.0028 | 1.0000 | -0.0014 | 0.8817 | -0.0022 | 1.0000 | 0.0040 | 0.1691 | 0.0017 | 0.1750 | 0.0006 | 0.3979 | 0.0277 | 0.0010 | 0.0094 | 0.0088 | -0.0013 | 1.0000 | -0.0027 | 1.0000 | 0.0013 | 0.2776 |
| HLD111 | 0.2543 | 0.0000 | 0.4404 | 0.0000 | 0.2742 | 0.0000 | 0.3067 | 0.0000 | 0.2328 | 0.0000 | 0.1858 | 0.0000 | 0.1293 | 0.0000 | 0.1564 | 0.0000 | 0.1214 | 0.0000 | 0.2006 | 0.0000 | 0.1783 | 0.0000 | 0.0017 | 0.3118 | -0.0008 | 0.5728 | 0.0796 | 0.0000 | 0.0848 | 0.0000 | 0.0680 | 0.0000 | 0.0595 | 0.0000 | 0.1154 | 0.0000 | 0.0757 | 0.0000 | 0.0477 | 0.0000 | 0.1238 | 0.0000 | 0.0543 | 0.0000 | 0.0927 | 0.0000 | 0.0881 | 0.0000 |
| HLD114 | 0.0742 | 0.0000 | 0.0242 | 0.0049 | 0.0359 | 0.0020 | 0.0639 | 0.0000 | 0.0237 | 0.0215 | 0.0346 | 0.0010 | -0.0026 | 1.0000 | 0.0000 | 0.5122 | 0.0077 | 0.1085 | 0.0065 | 0.0567 | 0.0103 | 0.0860 | -0.0023 | 1.0000 | -0.0027 | 1.0000 | 0.0277 | 0.0039 | 0.0445 | 0.0000 | 0.0336 | 0.0000 | 0.1042 | 0.0000 | 0.0419 | 0.0000 | 0.0586 | 0.0000 | 0.0469 | 0.0000 | 0.0326 | 0.0000 | 0.0152 | 0.0020 | 0.0538 | 0.0000 | 0.0650 | 0.0000 |
| HLD118 | -0.0043 | 1.0000 | 0.0284 | 0.0049 | -0.0044 | 1.0000 | 0.0030 | 0.2825 | 0.0018 | 0.3920 | -0.0042 | 1.0000 | 0.2177 | 0.0000 | 0.1560 | 0.0000 | 0.1976 | 0.0000 | 0.1345 | 0.0000 | 0.1883 | 0.0000 | 0.0522 | 0.0000 | 0.0127 | 0.0391 | 0.1044 | 0.0000 | 0.0927 | 0.0000 | 0.0918 | 0.0000 | 0.0661 | 0.0000 | 0.1605 | 0.0000 | 0.1114 | 0.0000 | 0.0409 | 0.0000 | 0.1693 | 0.0000 | 0.0984 | 0.0000 | 0.0636 | 0.0000 | 0.1584 | 0.0000 |
| HLD122 | 0.3013 | 0.0000 | 0.2690 | 0.0000 | 0.1895 | 0.0000 | 0.1856 | 0.0000 | 0.1651 | 0.0000 | 0.2345 | 0.0000 | 0.0160 | 0.0098 | 0.0002 | 0.4575 | 0.0046 | 0.2053 | 0.0468 | 0.0000 | -0.0053 | 1.0000 | -0.0015 | 0.8025 | -0.0022 | 1.0000 | 0.0129 | 0.0479 | -0.0028 | 1.0000 | 0.0012 | 0.3421 | 0.0191 | 0.0039 | 0.0228 | 0.0000 | 0.0465 | 0.0000 | 0.0858 | 0.0000 | 0.0113 | 0.0068 | 0.0074 | 0.0459 | 0.0163 | 0.0156 | 0.0240 | 0.0010 |
| HLD124 | 0.1346 | 0.0000 | 0.1384 | 0.0000 | 0.0733 | 0.0000 | 0.0936 | 0.0000 | 0.0689 | 0.0010 | 0.0863 | 0.0000 | -0.0026 | 1.0000 | 0.0157 | 0.0459 | -0.0047 | 1.0000 | -0.0018 | 1.0000 | -0.0034 | 1.0000 | 0.0351 | 0.0010 | 0.0067 | 0.1183 | 0.0139 | 0.0342 | 0.0179 | 0.0098 | 0.0870 | 0.0000 | 0.0253 | 0.0039 | 0.0236 | 0.0000 | 0.0382 | 0.0000 | 0.0372 | 0.0010 | 0.0176 | 0.0020 | 0.0070 | 0.0381 | 0.0042 | 0.1984 | 0.0083 | 0.0303 |
| HLD125 | 0.1323 | 0.0000 | 0.2925 | 0.0000 | 0.1439 | 0.0000 | 0.1613 | 0.0000 | 0.0647 | 0.0000 | 0.1261 | 0.0000 | 0.0006 | 0.4272 | 0.0005 | 0.3979 | -0.0020 | 0.8524 | 0.0010 | 0.2845 | -0.0035 | 1.0000 | -0.0027 | 1.0000 | -0.0014 | 0.7898 | 0.0017 | 0.2874 | 0.0043 | 0.1711 | 0.0102 | 0.0352 | 0.0017 | 0.3099 | 0.0390 | 0.0000 | 0.0256 | 0.0000 | 0.0168 | 0.0108 | 0.0398 | 0.0000 | 0.0144 | 0.0039 | 0.0077 | 0.0606 | 0.0411 | 0.0000 |
| HLD128 | 0.1701 | 0.0000 | 0.1635 | 0.0000 | 0.0867 | 0.0000 | 0.0805 | 0.0000 | 0.0587 | 0.0020 | 0.0419 | 0.0010 | 0.0064 | 0.0870 | 0.0016 | 0.3597 | 0.0065 | 0.1271 | 0.0203 | 0.0020 | 0.0289 | 0.0039 | 0.0020 | 0.3157 | -0.0020 | 1.0000 | 0.0031 | 0.2454 | 0.0136 | 0.0176 | 0.0084 | 0.0577 | 0.0327 | 0.0000 | 0.0077 | 0.0098 | 0.0015 | 0.2375 | 0.0071 | 0.1046 | 0.0007 | 0.3353 | -0.0013 | 1.0000 | 0.1145 | 0.0000 | 0.0001 | 0.4868 |
| HLD131 | 0.0081 | 0.1056 | 0.0441 | 0.0000 | 0.0481 | 0.0000 | 0.0628 | 0.0000 | 0.0085 | 0.1584 | -0.0043 | 1.0000 | 0.0075 | 0.0821 | 0.0058 | 0.1613 | -0.0005 | 0.5445 | 0.0286 | 0.0010 | 0.0047 | 0.2405 | -0.0003 | 0.4976 | -0.0038 | 1.0000 | 0.0088 | 0.1066 | -0.0031 | 1.0000 | 0.0034 | 0.2053 | -0.0015 | 1.0000 | 0.0228 | 0.0000 | 0.0268 | 0.0020 | 0.0213 | 0.0156 | 0.0160 | 0.0020 | 0.0384 | 0.0000 | 0.0532 | 0.0000 | 0.0274 | 0.0000 |
| HLD133 | 0.0352 | 0.0010 | 0.0540 | 0.0000 | 0.0600 | 0.0010 | 0.0269 | 0.0117 | 0.0166 | 0.0577 | 0.0180 | 0.0235 | 0.0163 | 0.0147 | 0.0473 | 0.0000 | 0.0598 | 0.0000 | 0.0312 | 0.0010 | 0.0528 | 0.0010 | -0.0024 | 1.0000 | -0.0036 | 1.0000 | 0.0059 | 0.1378 | -0.0027 | 1.0000 | 0.0032 | 0.2199 | 0.0036 | 0.1896 | 0.0134 | 0.0010 | 0.0167 | 0.0000 | 0.0332 | 0.0010 | 0.0079 | 0.0205 | 0.0010 | 0.3265 | 0.0266 | 0.0000 | 0.0119 | 0.0059 |
| HLD136 | 0.0076 | 0.1241 | 0.0487 | 0.0020 | -0.0047 | 1.0000 | -0.0034 | 1.0000 | 0.0111 | 0.1232 | -0.0022 | 1.0000 | 0.0164 | 0.0068 | -0.0022 | 1.0000 | 0.0129 | 0.0675 | -0.0015 | 1.0000 | 0.0996 | 0.0000 | 0.0020 | 0.3197 | -0.0023 | 1.0000 | 0.0180 | 0.0166 | 0.0228 | 0.0059 | 0.0133 | 0.0342 | 0.0018 | 0.3245 | 0.0059 | 0.0235 | -0.0016 | 1.0000 | 0.0280 | 0.0000 | 0.0266 | 0.0010 | 0.0107 | 0.0186 | 0.0215 | 0.0010 | 0.0064 | 0.0684 |

| Supplementary Table 3. *Fst* values between Kyrgyz group and other populations based on raw data of the same set of 30 DIP loci | | | | | | | | | | | | | | | | | | | | | | | | | |
| --- | --- | --- | --- | --- | --- | --- | --- | --- | --- | --- | --- | --- | --- | --- | --- | --- | --- | --- | --- | --- | --- | --- | --- | --- | --- |
| **Populations** | **Yucatan Mexican** | **Mexican Amerindian** | **Mexico Mexican** | **Veracruz Mexican** | **Jalisco Mexican** | **Chilhuahua Mexican** | **Uruguayan** | **Dane** | **Central Spanish** | **Hungarian** | **Basque** | **Uygur** | **Kyrgyz** | **Kazakh** | **Beijing Han** | **Tibet Tibetan** | **Qinghai Tibetan** | **Bai** | **Shanghai Han** | **Guangdong Han** | **She** | **South Korean** | **Xibe** | **Yi** | **Tujia** |
| Yucatan Mexican | * |  |  |  |  |  |  |  |  |  |  |  |  |  |  |  |  |  |  |  |  |  |  |  |  |
| Mexican Amerindian | 0.0128 | * |  |  |  |  |  |  |  |  |  |  |  |  |  |  |  |  |  |  |  |  |  |  |  |
| Mexico Mexican | 0.0082 | 0.0233 | * |  |  |  |  |  |  |  |  |  |  |  |  |  |  |  |  |  |  |  |  |  |  |
| Veracruz Mexican | 0.0044 | 0.0141 | 0.0038 | * |  |  |  |  |  |  |  |  |  |  |  |  |  |  |  |  |  |  |  |  |  |
| Jalisco Mexican | 0.0090 | 0.0207 | 0.0061 | 0.0003 | * |  |  |  |  |  |  |  |  |  |  |  |  |  |  |  |  |  |  |  |  |
| Chilhuahua Mexican | 0.0121 | 0.0247 | 0.0116 | 0.0092 | -0.0011 | * |  |  |  |  |  |  |  |  |  |  |  |  |  |  |  |  |  |  |  |
| Uruguayan | 0.1012 | 0.1347 | 0.0918 | 0.0761 | 0.0644 | 0.0723 | * |  |  |  |  |  |  |  |  |  |  |  |  |  |  |  |  |  |  |
| Dane | 0.0904 | 0.1176 | 0.0740 | 0.0651 | 0.0569 | 0.0635 | 0.0107 | * |  |  |  |  |  |  |  |  |  |  |  |  |  |  |  |  |  |
| Central Spanish | 0.0940 | 0.1262 | 0.0861 | 0.0664 | 0.0603 | 0.0688 | 0.0036 | 0.0055 | * |  |  |  |  |  |  |  |  |  |  |  |  |  |  |  |  |
| Hungarian | 0.0773 | 0.0987 | 0.0695 | 0.0538 | 0.0458 | 0.0544 | 0.0049 | 0.0059 | 0.0039 | * |  |  |  |  |  |  |  |  |  |  |  |  |  |  |  |
| Basque | 0.0943 | 0.1274 | 0.0862 | 0.0708 | 0.0661 | 0.0733 | 0.0107 | 0.0118 | 0.0054 | 0.0123 | * |  |  |  |  |  |  |  |  |  |  |  |  |  |  |
| Uygur | 0.1194 | 0.1531 | 0.1059 | 0.0905 | 0.0774 | 0.0845 | 0.0178 | 0.0273 | 0.0211 | 0.0226 | 0.0312 | * |  |  |  |  |  |  |  |  |  |  |  |  |  |
| Kyrgyz | 0.1460 | 0.1742 | 0.1290 | 0.1150 | 0.1008 | 0.1092 | 0.0314 | 0.0387 | 0.0366 | 0.0388 | 0.0458 | 0.0057 | * |  |  |  |  |  |  |  |  |  |  |  |  |
| Kazakh | 0.1328 | 0.1668 | 0.1170 | 0.1039 | 0.0895 | 0.0973 | 0.0214 | 0.0310 | 0.0271 | 0.0283 | 0.0368 | 0.0000 | 0.0003 | * |  |  |  |  |  |  |  |  |  |  |  |
| Beijing Han | 0.1921 | 0.2259 | 0.1757 | 0.1655 | 0.1457 | 0.1488 | 0.0725 | 0.0813 | 0.0845 | 0.0801 | 0.0887 | 0.0276 | 0.0145 | 0.0220 | * |  |  |  |  |  |  |  |  |  |  |
| Tibet Tibetan | 0.1996 | 0.2341 | 0.1799 | 0.1673 | 0.1520 | 0.1552 | 0.0662 | 0.0792 | 0.0767 | 0.0755 | 0.0863 | 0.0253 | 0.0139 | 0.0184 | 0.0078 | * |  |  |  |  |  |  |  |  |  |
| Qinghai Tibetan | 0.1930 | 0.2261 | 0.1757 | 0.1629 | 0.1460 | 0.1503 | 0.0647 | 0.0777 | 0.0773 | 0.0748 | 0.0856 | 0.0249 | 0.0151 | 0.0199 | 0.0046 | 0.0010 | * |  |  |  |  |  |  |  |  |
| Bai | 0.1955 | 0.2255 | 0.1744 | 0.1635 | 0.1474 | 0.1523 | 0.0675 | 0.0778 | 0.0785 | 0.0761 | 0.0815 | 0.0255 | 0.0147 | 0.0226 | 0.0034 | 0.0056 | 0.0022 | * |  |  |  |  |  |  |  |
| Shanghai Han | 0.2000 | 0.2307 | 0.1841 | 0.1727 | 0.1557 | 0.1620 | 0.0818 | 0.0918 | 0.0921 | 0.0923 | 0.0937 | 0.0354 | 0.0198 | 0.0296 | 0.0014 | 0.0119 | 0.0061 | 0.0028 | * |  |  |  |  |  |  |
| Guangdong Han | 0.1996 | 0.2291 | 0.1845 | 0.1713 | 0.1551 | 0.1621 | 0.0825 | 0.0916 | 0.0919 | 0.0921 | 0.0930 | 0.0367 | 0.0210 | 0.0307 | 0.0038 | 0.0168 | 0.0105 | 0.0047 | 0.0009 | * |  |  |  |  |  |
| She | 0.2145 | 0.2493 | 0.2000 | 0.1867 | 0.1699 | 0.1758 | 0.0842 | 0.0932 | 0.0963 | 0.0941 | 0.0994 | 0.0417 | 0.0257 | 0.0349 | 0.0032 | 0.0178 | 0.0111 | 0.0086 | 0.0043 | 0.0032 | * |  |  |  |  |
| South Korean | 0.2078 | 0.2399 | 0.1916 | 0.1805 | 0.1647 | 0.1702 | 0.0839 | 0.0962 | 0.0952 | 0.0958 | 0.0961 | 0.0404 | 0.0225 | 0.0338 | 0.0060 | 0.0119 | 0.0060 | 0.0036 | 0.0019 | 0.0051 | 0.0070 | * |  |  |  |
| Xibe | 0.1910 | 0.2224 | 0.1752 | 0.1631 | 0.1461 | 0.1532 | 0.0682 | 0.0782 | 0.0768 | 0.0779 | 0.0817 | 0.0281 | 0.0117 | 0.0193 | 0.0050 | 0.0102 | 0.0044 | 0.0057 | 0.0045 | 0.0073 | 0.0091 | 0.0038 | * |  |  |
| Yi | 0.2182 | 0.2482 | 0.1980 | 0.1881 | 0.1709 | 0.1730 | 0.0922 | 0.1046 | 0.1066 | 0.1031 | 0.1107 | 0.0517 | 0.0322 | 0.0415 | 0.0183 | 0.0230 | 0.0188 | 0.0120 | 0.0139 | 0.0123 | 0.0172 | 0.0142 | 0.0177 | * |  |
| Tujia | 0.2079 | 0.2395 | 0.1918 | 0.1801 | 0.1631 | 0.1681 | 0.0812 | 0.0907 | 0.0912 | 0.0909 | 0.0942 | 0.0371 | 0.0193 | 0.0299 | 0.0015 | 0.0108 | 0.0059 | 0.0032 | 0.0002 | 0.0010 | 0.0035 | 0.0020 | 0.0032 | 0.0129 | * |

| Supplementary Table 4. *D_A_* distances between Kyrgyz group and other populations based on allelic frequencies of the same set of 30 DIP loci | | | | | | | | | | | | | | | | | | | | | | | | | |
| --- | --- | --- | --- | --- | --- | --- | --- | --- | --- | --- | --- | --- | --- | --- | --- | --- | --- | --- | --- | --- | --- | --- | --- | --- | --- |
| **P**opulations | **Yucatan Mexican** | **Mexican Amerindian** | **Mexico Mexican** | **Veracruz Mexican** | **Jalisco Mexican** | **Chilhuahua Mexican** | **Uruguayan** | **Dane** | **Central Spanish** | **Hungarian** | **Basque** | **Uygur** | **Kyrgyz** | **Kazakh** | **Beijing Han** | **Tibet Tibetan** | **Qinghai Tibetan** | **Bai** | **Shanghai Han** | **Guangdong Han** | **She** | **South Korean** | **Xibe** | **Yi** | **Tujia** |
| Yucatan Mexican | * |  |  |  |  |  |  |  |  |  |  |  |  |  |  |  |  |  |  |  |  |  |  |  |  |
| Mexican Amerindian | 0.0053 | * |  |  |  |  |  |  |  |  |  |  |  |  |  |  |  |  |  |  |  |  |  |  |  |
| Mexico Mexican | 0.0041 | 0.0087 | * |  |  |  |  |  |  |  |  |  |  |  |  |  |  |  |  |  |  |  |  |  |  |
| Veracruz Mexican | 0.0031 | 0.0064 | 0.0030 | * |  |  |  |  |  |  |  |  |  |  |  |  |  |  |  |  |  |  |  |  |  |
| Jalisco Mexican | 0.0046 | 0.0090 | 0.0041 | 0.0024 | * |  |  |  |  |  |  |  |  |  |  |  |  |  |  |  |  |  |  |  |  |
| Chilhuahua Mexican | 0.0048 | 0.0093 | 0.0048 | 0.0040 | 0.0017 | * |  |  |  |  |  |  |  |  |  |  |  |  |  |  |  |  |  |  |  |
| Uruguayan | 0.0300 | 0.0427 | 0.0274 | 0.0223 | 0.0189 | 0.0207 | * |  |  |  |  |  |  |  |  |  |  |  |  |  |  |  |  |  |  |
| Dane | 0.0267 | 0.0365 | 0.0222 | 0.0192 | 0.0170 | 0.0183 | 0.0039 | * |  |  |  |  |  |  |  |  |  |  |  |  |  |  |  |  |  |
| Central Spanish | 0.0279 | 0.0393 | 0.0258 | 0.0197 | 0.0181 | 0.0200 | 0.0023 | 0.0030 | * |  |  |  |  |  |  |  |  |  |  |  |  |  |  |  |  |
| Hungarian | 0.0230 | 0.0317 | 0.0210 | 0.0160 | 0.0137 | 0.0156 | 0.0021 | 0.0026 | 0.0022 | * |  |  |  |  |  |  |  |  |  |  |  |  |  |  |  |
| Basque | 0.0277 | 0.0389 | 0.0256 | 0.0209 | 0.0197 | 0.0212 | 0.0043 | 0.0048 | 0.0033 | 0.0045 | * |  |  |  |  |  |  |  |  |  |  |  |  |  |  |
| Uygur | 0.0351 | 0.0477 | 0.0313 | 0.0263 | 0.0225 | 0.0241 | 0.0057 | 0.0083 | 0.0069 | 0.0068 | 0.0096 | * |  |  |  |  |  |  |  |  |  |  |  |  |  |
| Kyrgyz | 0.0430 | 0.0549 | 0.0377 | 0.0328 | 0.0285 | 0.0306 | 0.0088 | 0.0108 | 0.0105 | 0.0107 | 0.0128 | 0.0023 | * |  |  |  |  |  |  |  |  |  |  |  |  |
| Kazakh | 0.0391 | 0.0519 | 0.0345 | 0.0301 | 0.0260 | 0.0278 | 0.0067 | 0.0093 | 0.0085 | 0.0084 | 0.0111 | 0.0013 | 0.0010 | * |  |  |  |  |  |  |  |  |  |  |  |
| Beijing Han | 0.0588 | 0.0718 | 0.0534 | 0.0498 | 0.0437 | 0.0445 | 0.0230 | 0.0251 | 0.0262 | 0.0255 | 0.0270 | 0.0100 | 0.0056 | 0.0083 | * |  |  |  |  |  |  |  |  |  |  |
| Tibet Tibetan | 0.0613 | 0.0752 | 0.0545 | 0.0501 | 0.0452 | 0.0460 | 0.0207 | 0.0242 | 0.0236 | 0.0237 | 0.0261 | 0.0091 | 0.0052 | 0.0072 | 0.0029 | * |  |  |  |  |  |  |  |  |  |
| Qinghai Tibetan | 0.0587 | 0.0718 | 0.0528 | 0.0483 | 0.0430 | 0.0442 | 0.0201 | 0.0235 | 0.0235 | 0.0232 | 0.0256 | 0.0089 | 0.0053 | 0.0075 | 0.0022 | 0.0012 | * |  |  |  |  |  |  |  |  |
| Bai | 0.0595 | 0.0716 | 0.0524 | 0.0485 | 0.0435 | 0.0449 | 0.0207 | 0.0233 | 0.0237 | 0.0234 | 0.0242 | 0.0089 | 0.0052 | 0.0080 | 0.0020 | 0.0023 | 0.0016 | * |  |  |  |  |  |  |  |
| Shanghai Han | 0.0598 | 0.0732 | 0.0544 | 0.0502 | 0.0448 | 0.0465 | 0.0240 | 0.0264 | 0.0268 | 0.0271 | 0.0270 | 0.0114 | 0.0063 | 0.0096 | 0.0011 | 0.0036 | 0.0021 | 0.0014 | * |  |  |  |  |  |  |
| Guangdong Han | 0.0599 | 0.0724 | 0.0548 | 0.0500 | 0.0450 | 0.0471 | 0.0244 | 0.0265 | 0.0269 | 0.0275 | 0.0268 | 0.0118 | 0.0067 | 0.0100 | 0.0019 | 0.0050 | 0.0034 | 0.0021 | 0.0006 | * |  |  |  |  |  |
| She | 0.0655 | 0.0796 | 0.0606 | 0.0555 | 0.0502 | 0.0525 | 0.0255 | 0.0275 | 0.0285 | 0.0289 | 0.0288 | 0.0133 | 0.0081 | 0.0112 | 0.0023 | 0.0059 | 0.0041 | 0.0033 | 0.0019 | 0.0015 | * |  |  |  |  |
| South Korean | 0.0633 | 0.0770 | 0.0576 | 0.0535 | 0.0484 | 0.0500 | 0.0258 | 0.0288 | 0.0288 | 0.0295 | 0.0287 | 0.0135 | 0.0077 | 0.0115 | 0.0024 | 0.0037 | 0.0023 | 0.0019 | 0.0008 | 0.0017 | 0.0028 | * |  |  |  |
| Xibe | 0.0569 | 0.0698 | 0.0517 | 0.0473 | 0.0420 | 0.0441 | 0.0203 | 0.0227 | 0.0226 | 0.0231 | 0.0236 | 0.0092 | 0.0041 | 0.0068 | 0.0022 | 0.0034 | 0.0019 | 0.0023 | 0.0015 | 0.0023 | 0.0032 | 0.0016 | * |  |  |
| Yi | 0.0675 | 0.0798 | 0.0602 | 0.0566 | 0.0512 | 0.0522 | 0.0286 | 0.0315 | 0.0323 | 0.0325 | 0.0328 | 0.0163 | 0.0102 | 0.0133 | 0.0054 | 0.0066 | 0.0056 | 0.0038 | 0.0040 | 0.0038 | 0.0051 | 0.0042 | 0.0052 | * |  |
| Tujia | 0.0632 | 0.0764 | 0.0576 | 0.0534 | 0.0480 | 0.0495 | 0.0250 | 0.0272 | 0.0277 | 0.0281 | 0.0281 | 0.0125 | 0.0068 | 0.0104 | 0.0013 | 0.0035 | 0.0023 | 0.0017 | 0.0004 | 0.0007 | 0.0019 | 0.0009 | 0.0015 | 0.0040 | * |


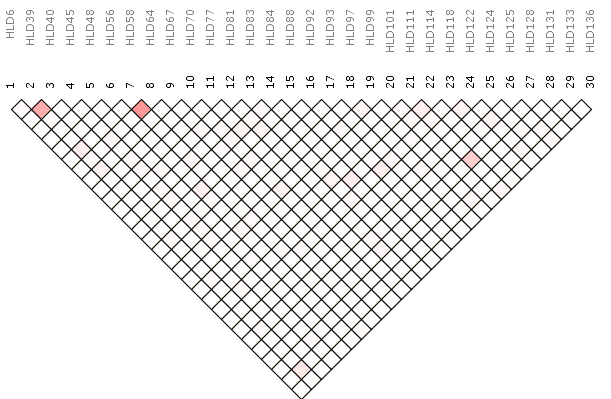


Supplementary Figure 1. Linkage disequilibrium analysis using the SNPAnalyzer program.


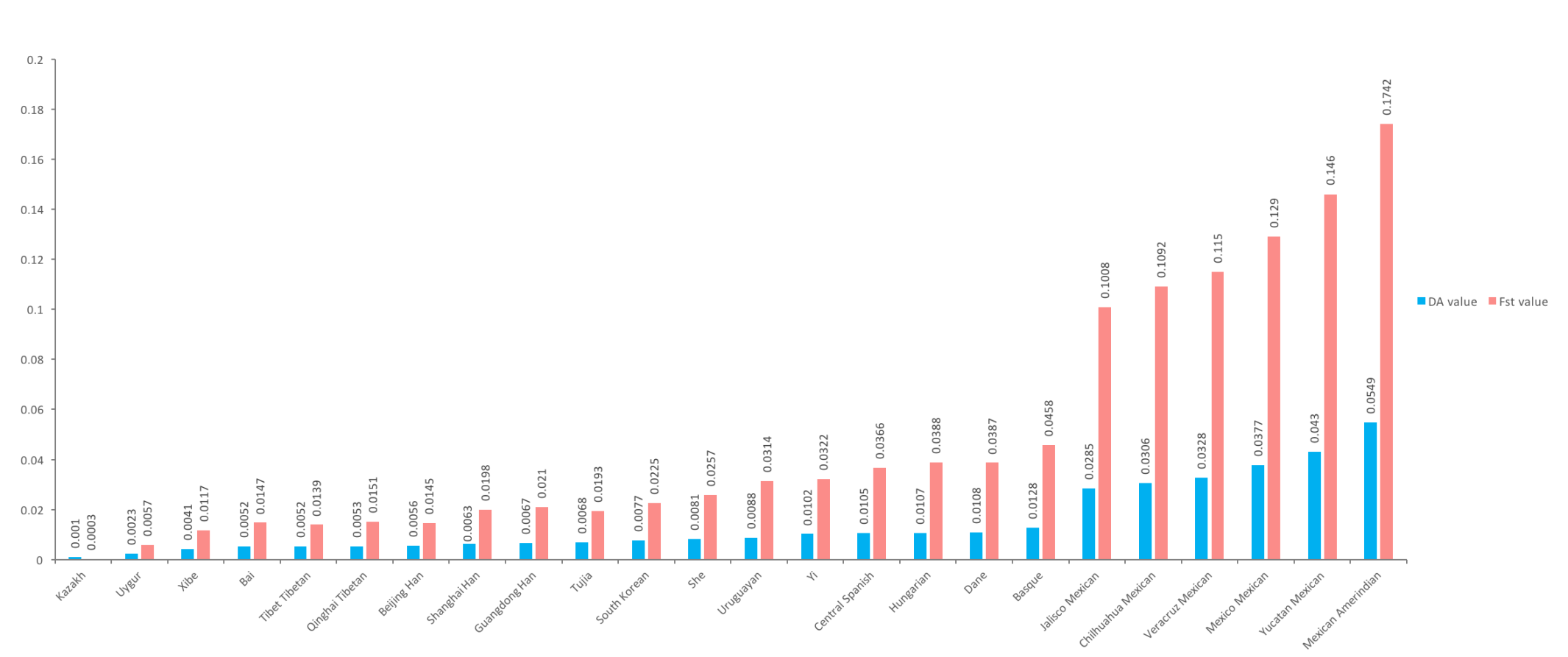


Supplementary Figure 2. Bar graph of both *Fst* and *D_A_* values between the studied Kyrgyz group and 24 compared groups.


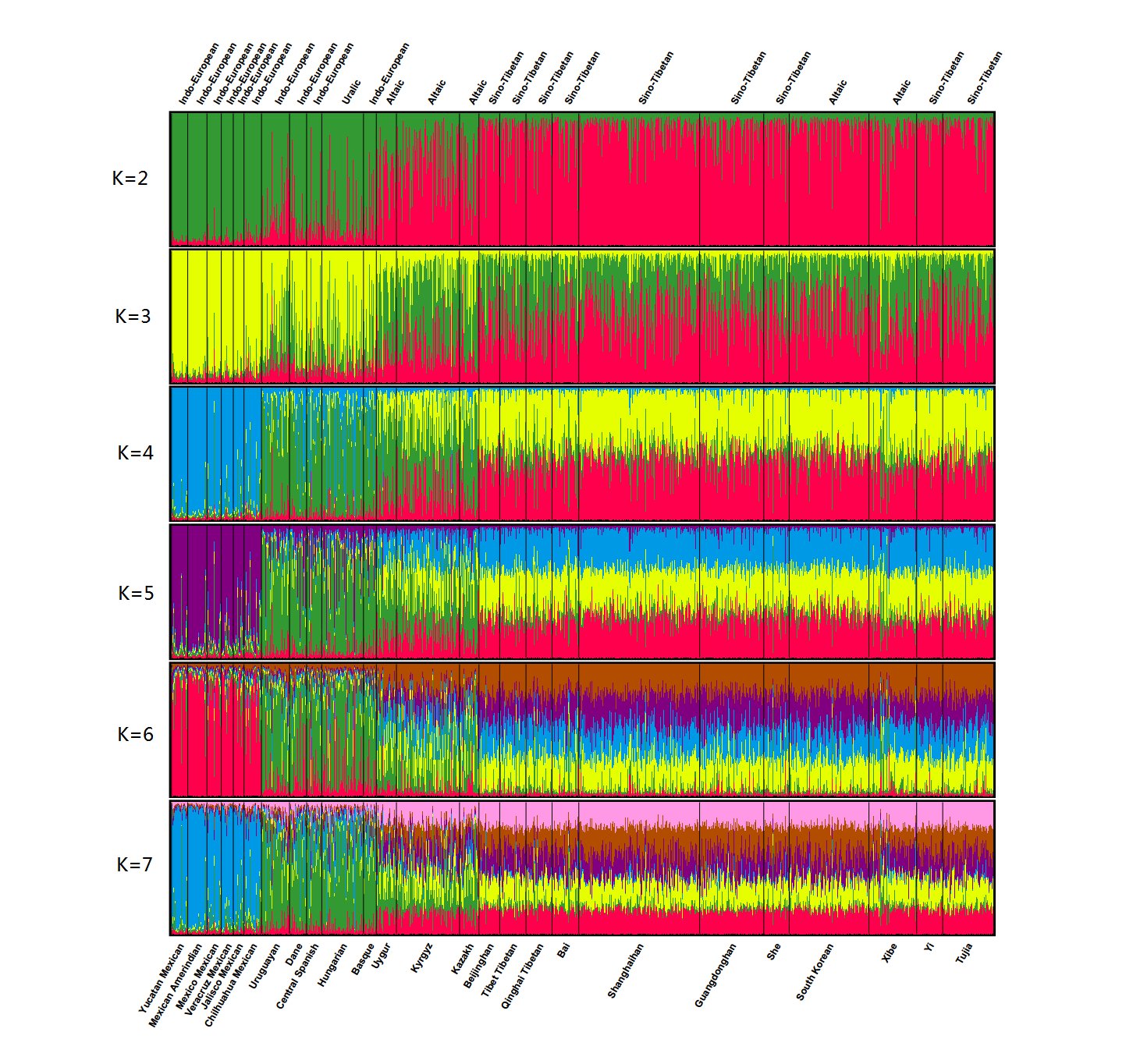


Supplementary Figure 3. STRUCTURE analysis at *K*=2-7 for all the individuals based on 30 DIP loci.


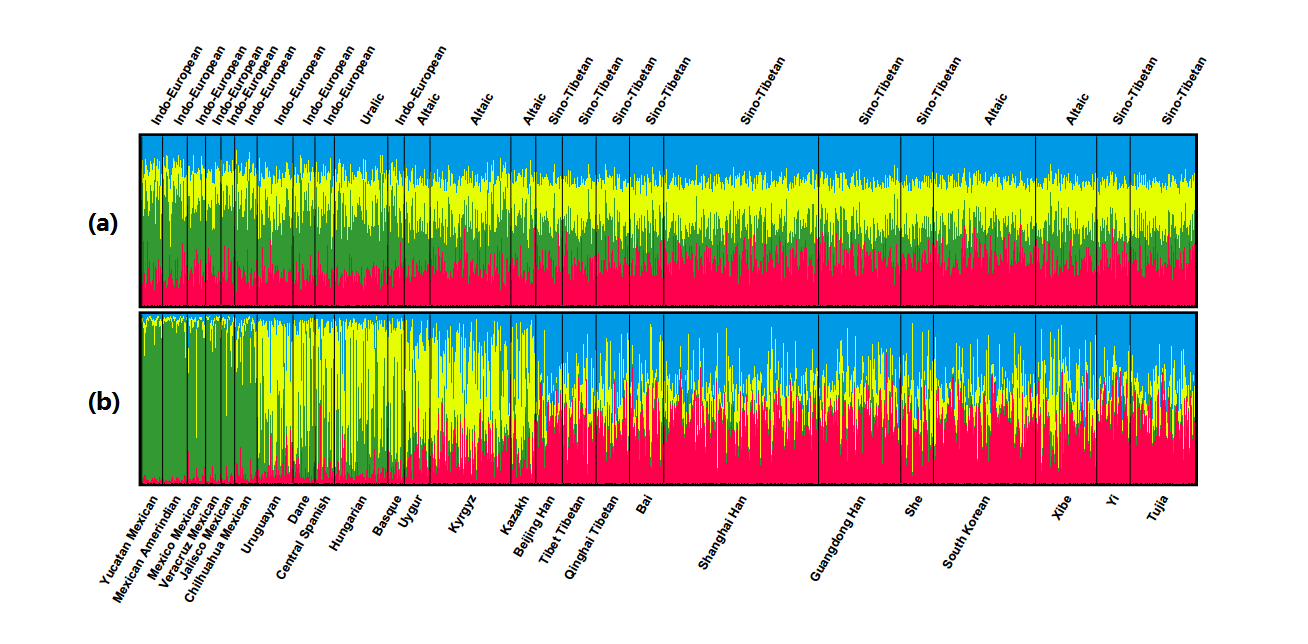


Supplementary Figure 4. STRUCTURE analysis of the studied Kyrgyz group and 24 referenced groups at *K*=4 (a) for all the individuals based on 15 eliminated loci; (b) for all the individuals based on 15 selected loci.
